# Impact of Three Different Mutations in *Ehrlichia chaffeensis* in Altering the Global Gene Expression Patterns

**DOI:** 10.1038/s41598-018-24471-3

**Published:** 2018-04-18

**Authors:** Chandramouli Kondethimmanahalli, Roman Ganta

**Affiliations:** Center of Excellence for Vector-Borne Diseases, Department of Diagnostic Medicine/Pathobiology, College of Veterinary Medicine, Kansas State University, Manhattan, Kansas 66506 USA

## Abstract

The rickettsial pathogen *Ehrlichia chaffeensis* causes a tick-borne disease, human monocytic ehrlichiosis. Mutations within certain genomic locations of the pathogen aid in understanding the pathogenesis and in developing attenuated vaccines. Our previous studies demonstrated that mutations in different genomic sites in *E*. *chaffeensis* caused variable impacts on their growth and attenuation in vertebrate and tick hosts. Here, we assessed the effect of three mutations on transcriptional changes using RNA deep-sequencing technology. RNA sequencing aided in detecting 66–80% of the transcripts of wildtype and mutant *E*. *chaffeensis*. Mutation in an antiporter gene (ECH_0379) causing attenuated growth in vertebrate hosts resulted in the down regulation of many transcribed genes. Similarly, a mutation downstream to the ECH_0490 coding sequence resulted in minimal impact on the pathogen’s *in vivo* growth, but caused major changes in its transcriptome. This mutation caused enhanced expression of several host stress response genes. Even though the ECH_0660 gene mutation caused the pathogen’s rapid clearance in vertebrate hosts and aids in generating a protective response, there was minimal impact on the transcriptome. The transcriptomic data offer novel insights about the impact of mutations on global gene expression and how they may contribute to the pathogen’s resistance and/or clearance from the host.

## Introduction

*Ehrlichia chaffeensis* is a tick-transmitted intracellular bacterial pathogen causing human monocytic ehrlichiosis (HME) and it also infects dogs, deer, goats, and coyotes^1–4^. Mutations at certain genomic locations, leading to gene expression changes, impact the pathogen’s ability to cause infection and persistence in a host^5,6^. The genome of *E*. *chaffeensis* may have evolved within a host cell environment leading to the development of mechanisms to undermine the host immune response^7^. Pathogenesis-associated *E*. *chaffeensis* genes are likely highly active in a host microenvironment and consistent with this hypothesis, differential gene expression in response to host cell defense is known to occur^8^. Progress has been made towards identifying genes crucial for *Ehrlichia* survival in a host cell environment^9–11^. However, to date only a few abundantly expressed genes are identified as associated with pathogenesis. Defining the genes involved in pathogenesis and virulence, and documenting their differential expression may aid in the discovery of novel proteins valuable as targets for therapeutic interventions and vaccine development for HME.

Genetically mutated intracellular pathogens are important resources for studying microbial pathogenesis, and also aid in the efforts of vaccine development^12,13^. Our previous study demonstrated the feasibility of transposon-based mutations in *E*. *chaffeensis*^5,6^. We also found that some insertion mutations resulting in transcriptional inactivation of membrane protein genes cause attenuation of the growth of the pathogen in vertebrate hosts. Insertions within the coding regions of ECH_0379 and ECH_0660 genes offered varying levels of protection against infection in a vertebrate host^14^. In this study, we hypothesized that the mutations’ specific genomic locations may impact global gene expression and contribute to the pathogen’s altered survival, infection progression, and replication in a host cell environment. To test this hypothesis, we assessed the impact of three mutations, reported earlier by Cheng *et al*.^5^, on global gene transcription. We selected two mutants with mutations within the coding regions of the ECH_0660 gene encoding for a phage like protein (ECH_0660) and the ECH_0379 gene encoding for an anti-porter protein (ECH_0379). Insertion mutation in ECH_0660 gene is located at the nucleotide position 213 of the 555 base long open reading frame. Similarly, mutation in ECH_0379 gene is located at the nucleotide position 682 of the 1056 base long open reading frame. The third insertion mutant strain, ECH_0490, has the insertion mutation 166 nucleotides downstream from the stop codon of ECH_0490 gene.

High throughput RNA sequencing (RNA seq) technologies have proven to be reliable and robust tools for determining global transcriptome activity in obligate intracellular bacteria^12,15–17^. Comparative genomic studies identified several classes of virulence factors involved in secretion and trafficking of molecules between the pathogen and host cells and modulation of the host immune response^18–20^. However, studies focused on *Ehrlichia* gene expression have been limited mostly to outer membrane proteins genes, Type IV Secretion System (T4SS) genes, tandem repeat protein (TRP) genes, and ankyrin repeat genes (Anks)^9,19,21–23^. Among them, genes encoding for T4SS proteins and p28-OMP proteins have been found to be critical for pathogenicity^9,24^.

The obligate intracellular nature of *E*. *chaffeensis* poses a challenge in obtaining cell-free *Ehrlichia* from host cells^25^. Technical constraints in isolating *Ehrlichia* RNA from highly abundant host RNA remains an impediment in profiling of pathogen transcripts^26^. To overcome this limitation, we used an effective cell lysis strategy followed by density gradient centrifugation. Further, we enriched *Ehrlichia* RNA by efficiently removing polyadenylated RNA (poly(A) RNA) and eukaryotic and prokaryotic ribosomal RNAs from host and bacteria RNA mixtures. Sequencing of the enriched RNA aided in the detection of transcripts for 66–80% of the annotated *E*. *chaffeensis* genes as per the annotated genome: GenBank #CP000236.1. Comparison of transcript levels from wildtype and mutant strains revealed the highest degree of modulation in immunogenic and secretory protein genes, particularly in the mutant strains of ECH_0490 and ECH_0379, while minimal changes were observed in the ECH_0660 mutant strain.

## Results

### Isolation and purification of cell-free *E*. *chaffeensis* from host cells

The major challenge of undertaking transcriptome studies of intracellular pathogens is the difficulty in isolating host-cell free bacteria and subsequently recovering high-quality bacterial RNA. Rickettsial organisms, including *E*. *chaffeensis*, constitute only a very small fraction of isolated total RNA^27,28^. Because of the presence of highly abundant host cell RNA, recovery of bacterial RNA is a challenge for executing RNA seq analysis experiments. In this study, we first purified the host cell-free bacteria from infected host cells (canine macrophage cell line, DH82) by employing an efficient cell lysis method, coupled with density gradient centrifugation protocols. Host cell lysis was performed to efficiently rupture the host cells without causing a major damage to the bacteria. *E*. *chaffeensis* organisms are about 0.5 to 1 µm in diameter. Therefore, infected host cell lysate was filtered through 2 µm membrane to remove most of the host cell debris. A high-speed Renografin density gradient centrifugation of the resulting *E*. *chaffeensis* cell suspension aided in pelleting bacteria while host cell debris remained at the top layer of the solution. After total RNA isolation and DNase treatment, Bioanalyzer analysis revealed that despite the prior fractionation of host cell-free bacteria, the host 28 S and 18 S RNA remained at high concentrations in the recovered RNA. Bacterial mRNA enrichment was carried out by depleting the host poly(A) RNA and eukaryotic ribosomal RNA using a bacterial RNA enrichment protocol, resulting in nearly undetectable levels of host 28 S and 18 S RNA (Supplementary Figures; Fig. S1 and Fig. S2). The absence of contaminating *E*. *chaffeensis* genomic DNA in the purified RNA samples was confirmed by real-time quantitative PCR using *E*. *chaffeensis* 16 S rRNA gene primers^27^. We also confirmed the absence of DNA sequences in the RNA seq raw data by aligning 20 randomly selected *E*. *chaffeensis* intergenic non-coding DNA sequences (data not shown).

### Ubiquitous transcription of genes in *E*. *chaffeensis* mutants

**Figure 1:**
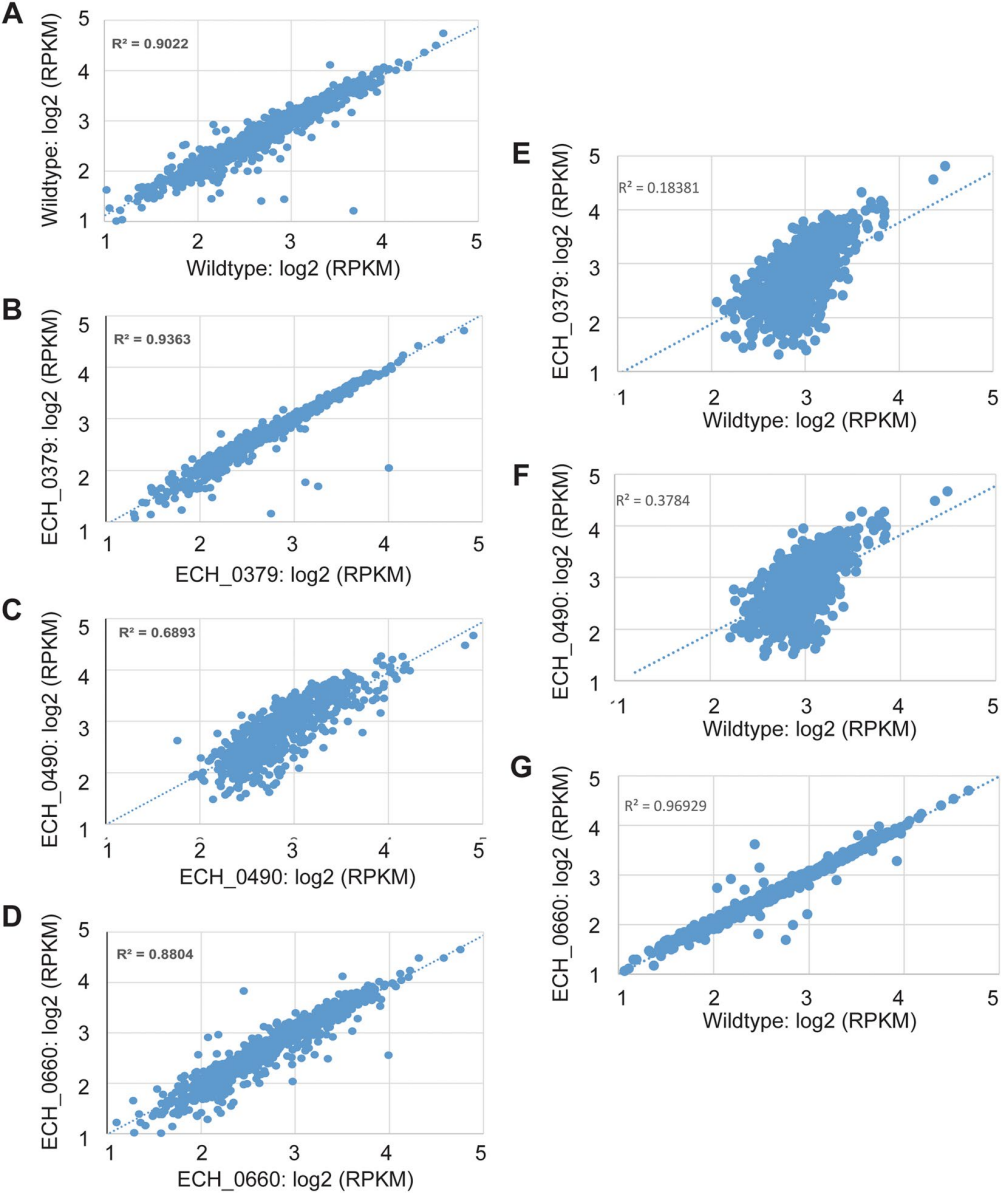
Scatter plot of RNA seq expression analysis. Scatter plots of transcript expression data for replicates of *E*. *chaffeensis* wildtype (**A**) and mutants ECH_0379 (**B**), ECH_0490 (**C**), and ECH_066 (**D**) showing a high degree of correlation. Scatter plots of transcript expression data for wildtype vs. mutants: (**E**) wildtype vs. ECH_0379, (**F**) wildtype vs. ECH_0490, and (**G**) wildtype vs. ECH_0660. Transcripts with ≥ 1 FPKM and minimum of 10 mapped reads were used. The plot is on a log-transformed scale.

Illumina HiSeq. 4000 RNA seq of *E*. *chaffeensis* wildtype and mutants generated between 75–130 million reads. The transcriptome data were deposited in the NCBI Bio-Project ID:PRJNA428837 and SRA accession:SRP128532 (https://www.ncbi.nlm.nih.gov/sra/SRP128532). Despite efficient depletion of host ribosomal RNA, only a fraction (less than19%) of reads were mapped to *E*. *chaffeensis* genomes. Mapping of reads (10 reads minimum/gene) identified about 66–80% of the genes being expressed from the *Ehrlichia* genome as per the annotated genome (GenBank # CP000236.1); the transcriptome of wildtype organisms (n = 3) contained transcripts for about 920 genes of the total of 1158 genes, and similarly 888, 895, and 768 gene transcripts (n = 3) were identified in mutant organisms ECH_0660, ECH_0379, and ECH_0490, respectively (Table 1). Table S1 lists total numbers of genes, and the expression value of the genes identified in the wildtype and all three mutant organisms. The replicate RNA seq data of wildtype (R² = 0.9) (Fig. 1A) and mutants ECH_0379 (R² = 0.93), (Fig. 1B), ECH_0490 (R² = 0.68) (Fig. 1C), and ECH_0660 (R² = 0.89) (Fig. 1D) showed a high degree of expression correlation. The scatter plot expression data of wildtype vs. ECH_0379 (R² = 0.18) (Fig. 1E) and wildtype vs. ECH_0490 (R² = 0.38) (Fig. 1F) showed a negative correlation. Notably, the expression plot of wildtype vs. ECH_0660 showed a positive correlation (R² = 0.96) (Fig. 1G). Only transcripts with reads per kilobase transcriptome per million mapped reads (RPKM) ≥ 1 were considered for differential expression analysis.

**Table 1:** Number of *E*. *chaffeensis* genes identified in three replicates of wildtype and its mutants.

No. of genes identified (>3 RPKM, 10 reads minimum)				
	Replicate 1	Replicate 2	Replicate 3	Avg (std dev)
Wildtype	888	900	973	920 (46)
ECH_0379	920	882	883	895 (21)
ECH_0490	841	670	793	768 (88)
ECH_0660	780	917	969	888 (97)

### Global transcriptome of *E*. *chaffeensis*

**Figure 2:**
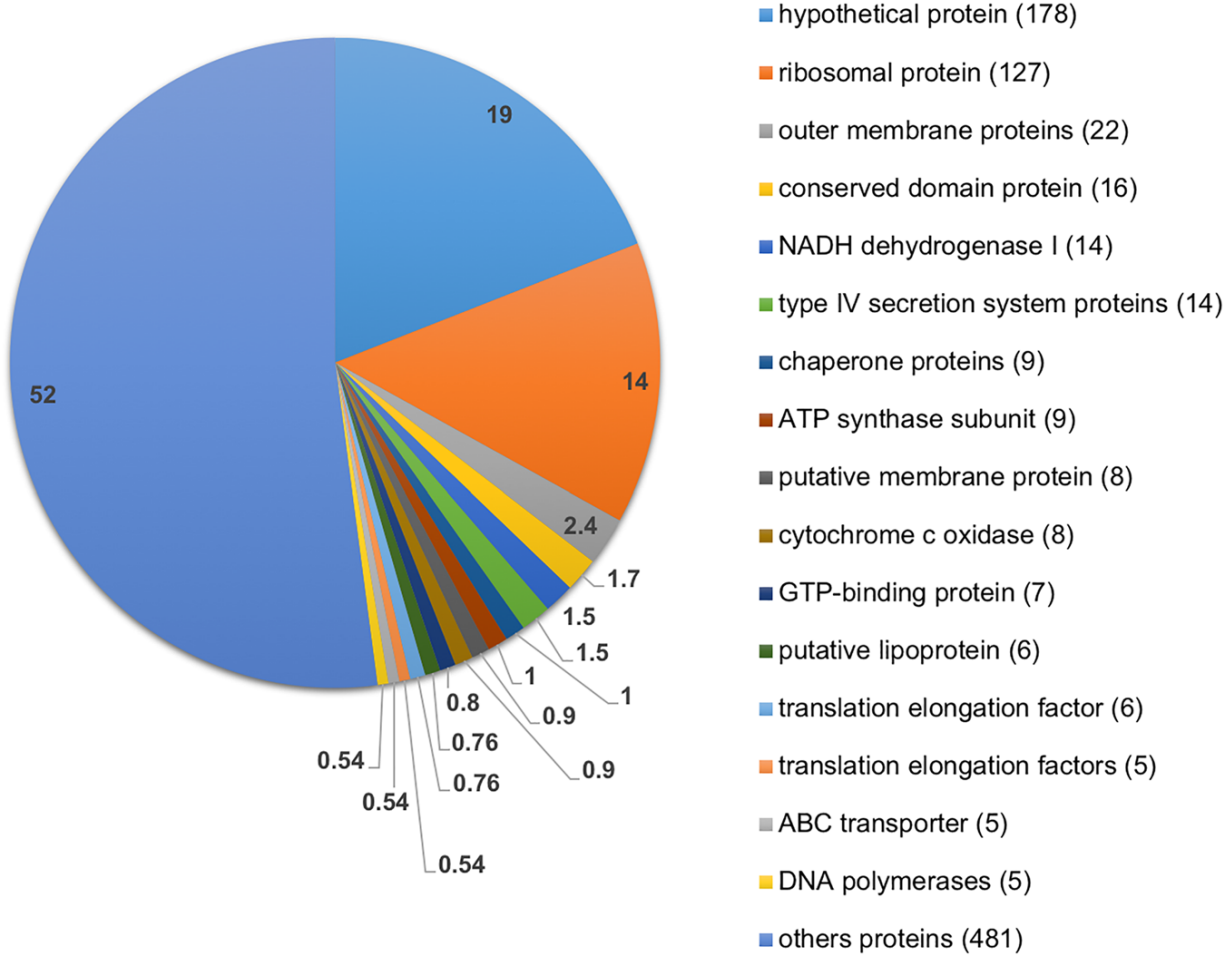
Distribution of the identified transcripts in wildtype *E*. *chaffeensis*. The inlaid numbers represent the percentage of transcripts detected in the RNA seq data (n = 3). The number of identified transcripts associated with each gene category is shown in the brackets. The minimum transcripts representation for each gene category was set to 5. The total number of genes identified was 920.

Distribution of the transcripts in wildtype *E*. *chaffeensis* (Fig. 2) included 481 transcripts represented by less than five transcripts, followed by hypothetical protein transcripts (178) representing 19% of transcriptome, and 127 ribosomal protein gene transcripts (14%). Transcripts of major outer membrane proteins (22 transcripts) represent the next most abundant group. Conserved domain protein transcripts encoded from 14 genes are associated with NADH dehydrogenase I complex. Other highly expressed genes included molecular chaperones, ATP synthase, putative membrane protein, cytochrome c oxidase, GTP-binding protein, putative lipoprotein, translation elongation factor, ABC transporter, and DNA polymerases; all of which represented 0.5–1.7% of the transcriptome. Table S2 lists the top 100 highly expressed genes in transcriptome of wildtype *E*. *chaffeensis*.

#### ECH_0379 mutation caused transcriptional down-regulation of many genes involved in antiporter activity, phage proteins, and those involved in transport and transcription function

Differential gene expression (DGE) was determined by comparing the RPKM expression values of mutants and wildtype (Fig. S3). Fold changes were considered significant with a p-value < 0.05, False Discovery Rate (FDR) ≤ 0.001, and consistency of expression values between replicates. The change in gene expression was not significant between wildtype and mutants for housekeeping genes. Based on these criteria, 41 genes were identified as predominantly downregulated and two genes were upregulated in the ECH_0379 gene mutant compared to wildtype (Table 2). The most prominent genes that showed a significant decrease in the transcription levels were those encoding for antiporter proteins, ABC transporters, and ATP-dependent Clp protease (ECH_0367). Four antiporter protein genes: monovalent cation/proton antiporter (ECH_0466), Na(+)/H(+) antiporter subunit C (mrpC) (ECH_0469), potassium uptake protein TrkH (ECH_1093), and nitrogen regulation protein NtrY (ECH_0299) showed a significant decline in the transcript levels. In addition, transcripts for two membrane transporters: cation ABC transporter permease protein transcript of the gene ECH_0517 and another ABC transporter permease protein transcript of the gene ECH_0972 were downregulated. Three genes coding for phage-like proteins {phage prohead protease (ECH_0032), phage portal protein (ECH_0033), and phage major capsid protein (ECH_0830)} were also downregulated in the mutant strain. Transcripts for 6 genes involved in transcription, namely DNA replication and repair protein RecF (ECH_0076), formamidopyrimidine-DNA glycosylase (ECH_0602), dimethyladenosine transferase (ECH_0648), GTP-binding protein EngA (ECH_0504), leucyl-tRNA synthetase (ECH_0794), and endonuclease III (ECH_0857) were also downregulated in this mutant strain. The enzymes of metabolic processes such as glutamate cysteine ligase (GCL) (ECH_0125), DNA/pantothenate metabolism flavoprotein (PMF) (ECH_0374), ATPase, AGF1 (ECH_0392), uroporphyrinogen III synthase (UPGS) (ECH_0480), diaminopimelate decarboxylase (DAPDC) (ECH_0485), biotin-acetyl-CoA-carboxylase ligase (BACL) (ECH_0848), and argininosuccinate lyase (ASL) (ECH_0937) are also down-regulated. Transcripts for 8 hypothetical protein genes; ECH_0021, ECH_0161, ECH_0264, ECH_0289, ECH_0725, ECH_0879, ECH_0913, and ECH_1053 were also among the downregulated genes in this mutant.

**Table 2:** *E*. *chaffeensis* genes differentially transcribed in ECH_0379 compared to wildtype.

Gene ID	Wildtype gene expression (RPKM)	ECH_0379 gene expression (RPKM)	Fold change (ECH_0379/Wildtype) FDR ≤ 0.001, p-value <0.05	Gene name
**Down regulated genes**				
ECH_0021	391	211	−1.88	conserved hypothetical protein
ECH_0032	82	26	−3.2	phage prohead protease, HK97 family
ECH_0033	41	20	−1.53	phage portal protein, HK97 family
ECH_0076	287	59	−5	putative DNA replication and repair protein RecF
ECH_0125	386	185	−2.08	glutamate-cysteine ligase
ECH_0161	81	42	−1.92	hypothetical protein
ECH_0188	586	121	−5	putative surface protein
ECH_0264	814	194	−4.16	conserved hypothetical protein
ECH_0289	102	52	−1.96	hypothetical protein
ECH_0299	1432	442	−1.81	putative nitrogen regulation protein NtrY
ECH_0367	3407	1784	−1.92	ATP-dependent Clp protease, ATP-binding subunit ClpB
ECH_0374	411	157	−2.63	DNA/pantothenate metabolism flavoprotein family protein
ECH_0392	845	159	−5.55	ATPase, AFG1 family
ECH_0466	432	252	−1.72	monovalent cation/proton antiporter
ECH_0469	137	52	−5.55	Na(+)/H(+) antiporter subunit C
ECH_0473	793	306	−5.55	aromatic-rich protein family
ECH_0480	319	92	−3.22	uroporphyrinogen-III synthase
ECH_0485	537	172	−3.14	diaminopimelate decarboxylase
ECH_0504	859	288	−3.03	GTP-binding protein EngA
ECH_0517	503	52	−10	putative cation ABC transporter, permease protein
ECH_0523	1525	159	−10	conserved domain protein
ECH_0541	251	124	−2	5-formyltetrahydrofolate cyclo-ligase family protein
ECH_0602	84	24	−3.57	formamidopyrimidine-DNA glycosylase
ECH_0648	399	138	−2.94	dimethyladenosine transferase
ECH_0725	648	280	−2.32	conserved hypothetical protein
ECH_0756	815	153	−5.55	divalent ion tolerance protein CutA1
ECH_0789	1154	363	−3.22	cytochrome c-type biogenesis protein CcmE
ECH_0794	1593	306	−5.26	leucyl-tRNA synthetase
ECH_0830	397	123	−3.22	phage major capsid protein, HK97 family
ECH_0848	1015	253	−4	biotin—acetyl-CoA-carboxylase ligase
ECH_0857	638	311	−2.04	endonuclease III
ECH_0864	455	246	−1.85	conserved domain protein
ECH_0879	520	153	−3.44	hypothetical protein
ECH_0913	570	114	−5	conserved hypothetical protein
ECH_0937	521	284	−1.85	argininosuccinate lyase
ECH_0972	524	285	−1.85	ABC transporter, permease protein
ECH_0998	722	332	−2.17	ubiquinone/menaquinone biosynthesis methlytransferase UbiE
ECH_1053	541	248	−2.22	conserved hypothetical protein
ECH_1063	201	106	−1.92	modification methylase, HemK family
ECH_1081	310	78	−4	SURF1 family protein
ECH_1084	684	364	−1.88	AraM protein
ECH_1093	973	320	−2.32	putative potassium uptake protein TrkH
ECH_1101	1143	190	−6.25	prolipoprotein diacylglyceryl transferase
**Up regulated genes**				
ECH_0684	1765	3651	2.06	ankyrin repeat protein
ECH_0495	942	1492	1.58	type IV secretion system protein VirB4

#### Differential transcriptional regulation of T4SS and p-28 OMP gene cluster genes in mutant ECH_0490

In the ECH_0490 mutant strain, 37 genes were significantly downregulated and 17 genes were up-regulated (Table 3). Four of the downregulated genes belonged to the T4SS are ECH_0494 (VirB3), ECH_0496 (VirB6), ECH_0498 (VirB6), and ECH_0499 (VirB6); and a type I secretion membrane fusion protein (T1SS_HlyD) (ECH_0970). Molecular chaperone genes, such as a cold shock protein (CSP) (ECH_0298) and ATP-dependent Clp protease, and a ATP-binding subunit ClpA (ClpA) were also downregulated. The transport proteins including the protein export membrane protein (SecF) (ECH_0095), preprotein translocase (SecY) (ECH_0428), potassium uptake protein (TrkH) (ECH_1093), and nitrogen regulation protein (NtrY) (ECH_0299) were also among the downregulated genes. Metabolic enzymes involved in biosynthetic processes, {tetrahydropyridine-2-carboxylate N-succinyltransferasem (dapD) (ECH_0058), quinone oxidoreductase (ECH_0385), metalloendopeptidase, (MEP) (ECH_0644), peptide deformylase (PDF) (ECH_0939), serine/threonine phosphatase (PSP) (ECH_0964), pyrophosphatase (PPi) (ECH_1014), and orotate phosphoribosyltransferase (OPRTase) (ECH_1108)}, were also down-regulated. Transcription- and translation-related genes, such as elongation factors (EF-Tu) (ECH_0515), aminoacyl-tRNA synthetases (IARS) (ECH_0538), DNA-binding protein (HU) (ECH_0804), 3′-5′ exonuclease domain (ECH_1011), and DNA-binding response regulator (ECH_1012), were also downregulated.

**Table 3:** *E*. *chaffeensis* genes differentially transcribed in ECH_0490 compared to wildtype.

Gene ID	Wildtype gene expression (RPKM)	ECH_0490 gene expression (RPKM)	Fold change (ECH_0490/wildtype) FDR ≤ 0.001, p-value <0.05	Gene name
**Down regulated genes**				
ECH_0058	1902	1006	−1.88	2,3,4,5-tetrahydropyridine-2-carboxylate N-succinyltransferase
ECH_0085	1119	523	−2.17	ABC transporter, ATP-binding protein
ECH_0095	1921	990	−1.96	protein-export membrane protein SecF
ECH_0264	814	310	−5.55	conserved hypothetical protein
ECH_0298	8295	3870	−2.17	cold shock protein, CSD family;
ECH_0299	719	314	−2.17	putative nitrogen regulation protein NtrY
ECH_0300	557	283	−2	putative ribonuclease D
ECH_0385	1659	663	−2.5	quinone oxidoreductase
ECH_0428	979	425	−2.32	preprotein translocase, SecY subunit
ECH_0470	1220	598	−2	ribonuclease, Rne/Rng family
ECH_0475	977	444	−2.22	signal recognition particle protein
ECH_0483	158	77	−2.04	primosomal protein N
ECH_0494	2326	1034	−2.17	type IV secretion system protein VirB3
ECH_0496	1059	435	−2.43	type IV secretion system protein VirB6
ECH_0498	1154	490	−2.38	type IV secretion system protein,VirB6 family
ECH_0499	1129	558	−2	type IV secretion system protein,VirB6 family
ECH_0515	1968	910	−2.17	translation elongation factor Ts
ECH_0525	1055	427	−2.5	conserved domain protein
ECH_0538	729	355	−2.08	isoleucyl-tRNA synthetase
ECH_0567	626	177	−3.57	ATP-dependent Clp protease, ATP-binding subunit ClpA
ECH_0585	475	229	−2.08	conserved domain protein
ECH_0644	1902	764	−2.5	putative metalloendopeptidase, glycoprotease family
ECH_0700	2670	1073	−2.5	hypothetical protein
ECH_0804	3113	1292	−2.43	DNA-binding protein HU
ECH_0820	409	167	−2.5	conserved hypothetical protein
ECH_0840	935	296	−3.22	2-polyprenylphenol 6-hydroxylase
ECH_0939	752	276	−2.77	putative polypeptide deformylase
ECH_0953	2914	1480	−2	ribosomal protein L7/L12
ECH_0964	1281	557	−2.32	serine/threonine phosphoprotein phosphatase
ECH_0970	474	247	−1.92	type I secretion membrane fusion protein, HlyD family
ECH_1011	2253	1104	−2.04	3′-5′ exonuclease family protein
ECH_1012	3353	1605	−2.08	DNA-binding response regulator
ECH_1014	1661	536	−3.12	inorganic pyrophosphatase
ECH_1093	973	416	−2.38	putative potassium uptake protein TrkH
ECH_1108	1903	938	−2.04	orotate phosphoribosyltransferase
ECH_1139	545	285	−1.92	major outer membrane protein OMP-1D
**Up-regulated genes**				
ECH_0009	7047	16828	2.38	putative membrane protein
ECH_0039	316	931	2.94	120 kDa immunodominant surface protein
ECH_0166	42488	96364	2.26	conserved hypothetical protein
ECH_0167	718	2654	3.70	tryptophanyl-tRNA synthetase
ECH_0169	161	397	2.46	riboflavin biosynthesis protein RibD
ECH_0230	991	4109	4.15	putative membrane protein
ECH_0251	1042	2185	2.1	hypothetical protein
ECH_0303	1018	2856	2.80	BolA family protein
ECH_0367	849	1274	2.49	ATP-dependent Clp protease, ATP-binding subunit ClpB
ECH_0450	1261	3710	2.94	conserved hypothetical protein
ECH_0531	1363	11788	8.65	hypothetical protein
ECH_0630	732	1688	2.30	FeS cluster assembly scaffold IscU
ECH_0655	1840	2763	2.03	RNA polymerase sigma-32 factor
ECH_0753	1932	4153	2.15	conserved hypothetical protein
ECH_0818	374	1222	3.26	major facilitator family transporter
ECH_0878	217	1126	5.17	hypothetical protein
ECH_1121	1578	3132	3.1	major outer membrane protein Omp-1N
ECH_1136	698	8270	2.37	major outer membrane protein OMP-1B
ECH_1143	3957	8359	2.24	major outer membrane protein P28
ECH_1146	190	1100	6.73	major outer membrane protein P28-2

Upregulated protein genes in this mutant included 7 that belonged to the transmembrane protein category. Of these, four belonged to the p-28 OMP gene cluster {ECH_1143 (OMP-p28), ECH_1146 (OMP-p28-2), ECH_1136 (OMP-1B), and ECH_1121 (OMP-1N)}. In addition, two putative membrane protein genes (ECH_0009, ECH_0230) and an immunodominant surface protein gene (ECH_0039) were upregulated. Transcripts for the heat shock proteins ATP-dependent Clp protease, ClpA (ECH_0567) and ATP-binding chaperon, ClpB (ECH_0367), and the stress response-associated RNA polymerase sigma factor (RpoH) (ECH_0655) were also upregulated. Transcripts for two genes coding for iron sulfur proteins {BolA family protein (ECH_0303) and FeS cluster assembly scaffold (IscU) (ECH_0630)} were similarly up-regulated. We observed differential expression of six hypothetical protein genes, which included ECH_0166, ECH_0251, ECH_0450, ECH_0531, ECH_0753, and ECH_0878.

#### Mutation in ECH_0660 gene led to minimal transcriptional alterations

While we observed drastic gene expression changes in both ECH_0379 and ECH_0490 mutants, ECH_0660 mutant transcriptome showed minimal variations compared to wildtype; we observed only five genes as notably differentially expressed in this mutant (Table 4). The genes included nitrogen regulation protein (NtrY) (ECH_0299) and the ABC transporter permease protein (ECH_0972) as down-regulated genes, whereas the heme exporter protein CcmA (ECH_0295) and chaperonin (ECH_0364) were upregulated. We also identified several commonly differentially-expressed genes in ECH_0379 and ECH_0490 (Table 5). The ribonuclease D (ECH_0300) and potassium uptake protein (ECH_1093) were commonly down regulated in ECH_0379 and ECH_0490. T4SS protein VirB4 gene was down-regulated in ECH_0490 mutant, whereas this gene was up-regulated in ECH_0379 mutant. Contrary to this, ClpB was down-regulated in ECH_0379 mutant and upregulated in ECH_0490 mutant.

**Table 4:** *E*. *chaffeensis* genes differentially transcribed in ECH_0660 compared to wildtype.

Gene ID	Wildtype gene expression (RPKM)	ECH_0660 gene expression (RPKM)	Fold change (ECH_0660/Wildtype) FDR ≤ 0.001, p-value <0.05	Gene name
**Down regulated genes**				
ECH_0299	1432	720	−2	putative nitrogen regulation protein NtrY
ECH_0972	524	309	−1.69	ABC transporter, permease protein
**Up regulated genes**				
ECH_0295	336	631	1.87	putative heme exporter protein CcmA
ECH_0364	6801	12150	1.78	chaperonin, 10 kDa
ECH_1147	1982	4756	2.39	conserved hypothetical protein

**Table 5:** *E*. *chaffeensis* common differentially transcribed genes in mutants.

Gene ID	Wildtype gene expression (RPKM)	Mutant gene expression (RPKM)	Fold change FDR ≤ 0.001, p-value < 0.05	Gene name	mutants
**Down regulated genes**					
ECH_0299	1432	442	−1.81	putative nitrogen regulation protein NtrY	ECH_0379 ECH_0490
ECH_0264	814	310	−2.63	conserved hypothetical protein	ECH_0379 ECH_0490
ECH_0300	557	283	−2	putative ribonuclease D	ECH_0379 ECH_0490
ECH_0864	279	193	−1.44	conserved domain protein	ECH_0379 ECH_0490
ECH_1093	972	416	−1.81	putative potassium uptake protein TrkH	ECH_0379 ECH_0490
ECH_0495	833	517	−1.63	type IV secretion system protein VirB4	ECH_0490
ECH_0367	3407	1783	−1.92	ATP-dependent Clp protease, ATP-binding subunit ClpB	ECH_0379
ECH_0745	712	437	−1.63	conserved domain protein	ECH_0379
**Up regulated**					
ECH_0495	942	1492	1.58	type IV secretion system protein VirB4	ECH_0379
ECH_0367	849	1275	2.49	ATP-dependent Clp protease, ATP-binding subunit ClpB	ECH_0490
ECH_0745	547	920	1.68	conserved domain protein	ECH_0490

### Validation of RNA seq data by quantitative real-time reverse transcription PCR

**Figure 3:**
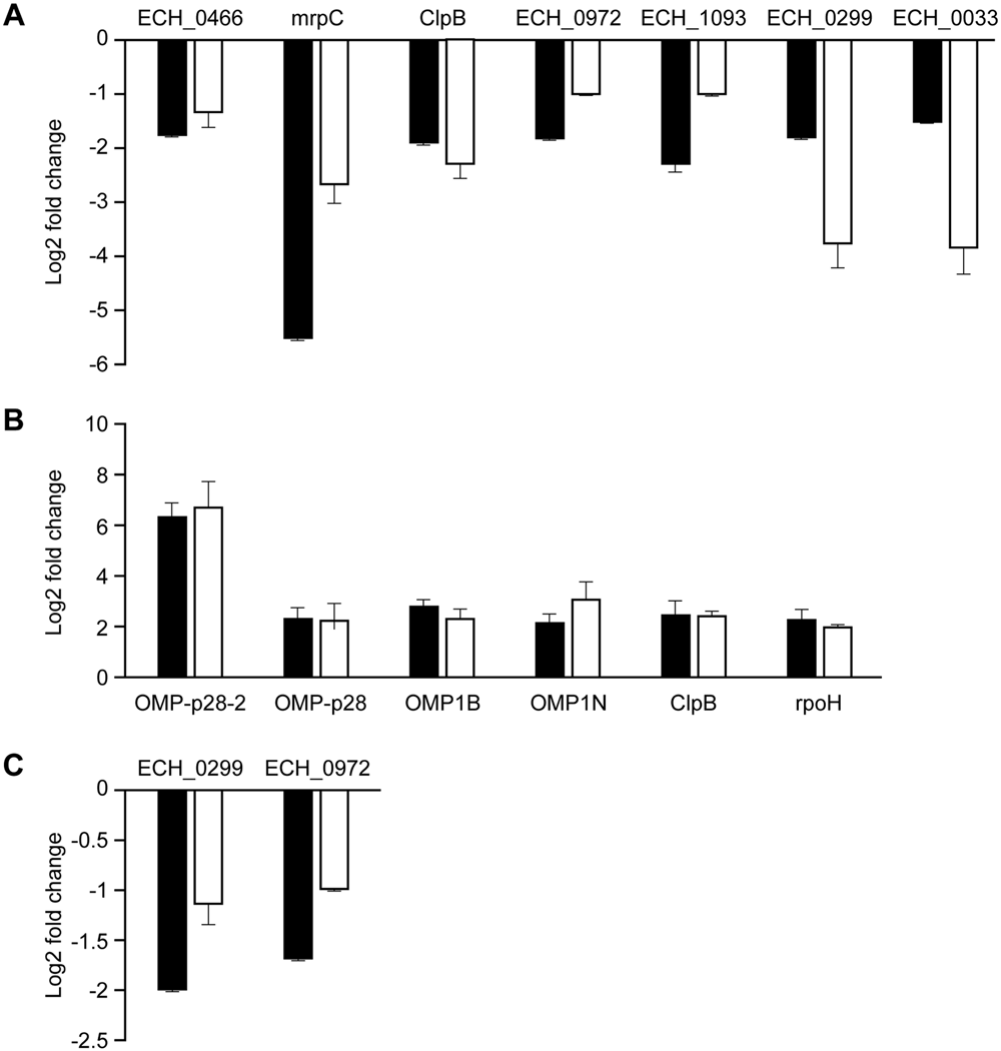
Verification of transcriptional variations observed in RNA seq data by qRT-PCR. Transcriptional fold changes in wildtype vs. ECH_0379 mutant (**A**), ECH_0490 mutant (**B**), or ECH_0660 mutant (**C**) were presented from the qRT-PCR data. Black bars represents RNA seq data and white bars represents qRT-PCR data.

Quantitative real-time quantitative reverse transcriptase-PCR (qRT-PCR) analysis was carried out on thirteen randomly selected genes identified as differentially transcribed according to the RNA seq data. To generate qRT-PCR data, we first normalized RNA samples to a constitutively expressed *E*. *chaffeensis* gene coding for the16S RNA as previously described in Cheng *et al*.^6^. The primers and genes selected for the qRT-PCR analysis are listed in Table S3. Transcript abundance for 7 down-regulated genes in ECH_379 mutant, including ECH_0466 and mrpC, ClpB, ECH_0033, NtrY, TrkH, and ECH_0972 were validated (Fig. 3A). Similarly, 6 upregulated genes from ECH_0490 mutant strain, including four transcripts belonging to an OMP gene cluster (OMP-p28, OMP-1B, OMP-1N, OMP-p28-2) and one each from ClpB and RpoH genes were verified by qRT-PCR (Fig. 3B). Likewise, the down-regulation of transcripts for the ECH_0299 and ECH_0972 genes were confirmed in ECH_0660 mutant by qRT-PCR (Fig. 3C).

## Discussion

Isolation of cell-free bacterial RNA from highly abundant host RNA is the first challenge in transcriptional profiling of intracellular pathogens^25,28,29^. Rickettsiales require culturing in host cells and then need to be purified before extracting RNA for transcriptome evaluation experiments. To document the impact of three transposon mutations on *E*. *chaffeensis* transcription, we first developed a method for isolation and purification of host cell-free *E*. *chaffeensis* organisms, from which we isolated RNA and then subjected to next generation sequencing (NGS) analysis. To isolate cell-free *E*. *chaffeensis*, we started with an efficient host cell lysis protocol, and then filtration of whole cell lysate, followed by a renografin density gradient centrifugation. The second challenge was to obtain host cell-free RNA for transcriptome profiling. Previous studies report that bacterial RNA enrichment methods result in the enrichment of bacterial RNA reads only 3–10%^29,30^. Isolation of host cell-free bacteria and the bacterial RNA purification steps implemented in our study allowed a greater enrichment of *E*. *chaffeensis* RNA. In our current studies, we were able to enrich the bacterial RNA, which helped in generating up to 19% high mapping RNA reads. Notably, deep RNA sequencing analysis aided in mapping 80% of *E*. *chaffeensis* genes expressed in infected macrophage host cells.

Among the highly expressed genes, the p28-OMP multigene cluster was dominant in the transcriptome. The *E*. *chaffeensis* p28-OMP multigene locus contains 22 tandemly arranged genes coding for the bacterial immunodominant proteins^31–33^. The presence of all 22 transcripts in the RNA seq data suggest that the gene cluster is among the most abundantly expressed genes. These observations are consistent with our previous proteomic study where we reported the p28*-*OMP genes’ expression abundance^33^. NADH dehydrogenase I complex genes were also highly expressed in *E*. *chaffeensis*. NADH dehydrogenase counters the phagosomal NOX2 response to inhibit host cell apoptosis^34^. T4SS effector proteins in some pathogenic bacteria are considered as important in manipulating a host gene expression to undermine the host immune response^35,36^. The contributions of T4SS effectors in pathogenicity are already reported for rickettsiales, including for *A*. *marginale*, *A*. *phagocytophilum*, *E*. *canis*, and *E*. *chaffeensis*^37–39^. The RNA seq analysis identified several transcripts encoding for T4SS proteins, including VirB3, B4, B6, B8, B9, B10, and B11. Chaperone protein genes DnaK, DnaJ, GroE, and ClpB were also highly expressed in both wildtype and mutant strains. The presence of such proteins involved in cell homeostasis and the oxidative stress response is reported in other rickettsiales^39–41^, suggesting that their gene products are also critical for the *E*. *chaffeensis* stress response if the pathogen proteome is similarly altered as per the transcriptome reported in the current study. Indeed, our recent study suggests that the stress response proteins are important for *E*. *chaffeensis*^11^. Other highly expressed protein genes included those encoding for house-keeping ribosomal proteins involved in protein synthesis, putative membrane proteins, ABC transporter, and lipoprotein; all of which are likely important for the pathogen’s protein synthesis, transport, trafficking, and effector secretion into the host cells. ATP synthase subunit, cytochrome c oxidase, DNA polymerases, GTP-binding protein and translation elongation factors involved energy metabolism, cell division, and transcriptional regulation were also among the highly expressed genes in both wildtype and mutant organisms. The extent of transcriptome coverage is higher than the previously reported for *E*. *chaffeensis* in ISE6 and AAE2 tick cells^8^. This is substantial for both the enhanced detection of intracellular pathogen transcripts and also because of the abundance of gene expressions observed. Higher coverage of the transcriptome likely resulted from deep sequencing of the RNAs by next-generation sequencing compared to microarray analysis^8^. This global set of highly expressed genes may represent products involved in pathogenicity, replication and survival of *E*. *chaffeensis* in host cell environment^42,43^. Four transcripts that code for ankyrin repeat proteins, which are shown to mediate protein-protein interactions^44^, were also identified in the transcriptome. Notably, the transcriptome from the wildtype and mutant organisms contained 216 transcripts that code for hypothetical proteins with unknown function. As these were within the core transcriptome, we anticipate that they represent an important set of transcribed genes for *E*. *chaffeensis* replication.

Transcription from large numbers of genes in ECH_0379 mutant was found to be reduced compared to wildtype. Genes representing antiporters, ABC transporters, chaperons, metabolic enzymes, and transcription regulators are among the down-regulated genes (Table 2). We predict that the mutation in the anti-porter protein gene caused a metabolic depression. Antiporter and transport proteins play an important role in the transport of ions and solutes across the cell membranes of bacteria^45^. Antiporters are integral membrane proteins that perform secondary transport of Na^+^ and/or K^+^ for H^+^ across a phospholipid membrane^5^. The *E*. *chaffeensis* genome contains several genes having homology to antiporter proteins or their subunits, suggesting that they are needed for the pathogen’s intraphagosomal replication and survival in a host. In particular, antiporters aid bacteria in maintaining pH, salt, and temperature conditions^46^. We observed a significant decline in transcription of antiporter genes such as monovalent cation/H + antiporter subunit C (ECH_0469) and ECH_0466. Disrupting the antiporter function or preventing their expression may affect the pathogen’s growth *in vivo*. Indeed, mutation in the ECH_0379 gene resulted in the attenuated growth of the organism in both an incidental host (dog) and in the reservoir host (white-tailed deer)^5,6^. ABC transporters also are involved in uptake of ions and amino acids and may play an important role in a pathogen’s ability to infect and survive in a host cell environment^47^. The ECH_0379 mutant had low levels of transcriptional activity of the genes ECH_0517 and ECH_0972 encoding for ABC transporters, which function at different stages in the pathogenesis of infection^47,48^. These proteins promote the survival of pathogens in the host microenvironments^49^. The mutation possibly interferes with transport mechanisms, thereby affecting its ability to infect and survive in host cells^5,6^. The mutation may have also caused alterations to the transcriptions of genes involved in physiological responses, such as regulating the pathogen’s metabolic activities. We also found down-regulation of several transcripts encoding for metabolic enzymes: glutamate–cysteine ligase, DNA/pantothenate metabolism flavoprotein family protein, ATPase, uroporphyrinogen-III synthase, diaminopimelate decarboxylase, biotin–acetyl-CoA-carboxylase ligase, and argininosuccinate lyase. In general, a pathogen’s survival in an intracellular environment depends on its ability to derive nutrients from the host cell^50^. Pathogenic bacteria use metabolic pathways and virulence-associated factors that undermine the host immune system so that they can derive nutrients from their host cells^51^. It is possible that the downregulation of the transcripts from the aforementioned genes in the ECH_0379 mutant hampers the bacterial metabolic response and its capacity to derive nutrients from the host. The mutation also caused decreased expression of genes encoding DNA replication and repair protein, formamidopyrimidine-DNA glycosylase, dimethyladenosine transferase, and leucyl-tRNA synthetase. This may have also contributed to defects in pathogen’s intracellular growth and survival. Our prior studies suggest that despite the mutant’s attenuated growth, it failed to offer complete protection against wildtype infection challenge^14^. If the changes in the transcriptome correlate with changes in the proteome, variations in the mutant organisms’ protein expression relative to the wildtype *E*. *chaffeensis* may result in an altered host response, thus making the host less effective in initiating a protective host response when exposed to the mutant organisms^14^.

Pathogenic bacteria produce T4SS effectors to weaken the host cell gene expression and contributes to bacterial virulence^52,53^. RNA seq data suggested declined expressions of various T4SS component protein gene transcripts in ECH_0490 mutant. We also observed decreased transcription of chaperone proteins and several genes involved in the transcription and translational machinery, and exonuclease and DNA-binding regulator gene transcripts in the ECH_0490 mutant strain. On the contrary, ClpB (a major stress response heat shock protein) and RpoH (stress response RNA polymerase transcriptional subunit) showed increased transcription in the mutant.

Chaperone proteins play a key role in protein disaggregation and in aiding the pathogen to overcome the likely host cell-induced stress^54^. ClpB reactivates aggregated proteins accumulating under stress conditions and it was abundantly expressed during replication stage of *E*. *chaffeensis*^54,55^. Preventing or reducing protein aggregation and the associated protein inactivation during the bacterial growth within a host cell may benefit the pathogen in enhancing its survival^11^. The RNA polymerase transcription regulator, RpoH, is also important for the pathogen’s continued growth as it aids in promoting the expression of stress response proteins^10^. Consistent with the prediction, increased expression of ClpB and RpoH was observed in the current study for ECH_0490 mutant. The enhanced expression from these two important genes likely enables the mutant to grow similarly to wildtype *E*. *chaffeensis* in vertebrate and tick hosts, as reported in our previous studies^5,6^. Outer membrane proteins perform a variety of functions such as invasion, transport, immune response, and adhesion that are vital to the survival of *Ehrlichia* species, including *E*. *chaffeensis* and *E*. *ruminantium* in a host^32,56–59^. The ECH_0490 mutant had increased abundance of OMPs compared to wildtype organisms. We found seven transmembrane genes coding for immunodominant P28/OMP family of proteins (OMP_p28, OMP_p28-2, OMP-1B, and OMP-1N) and membrane proteins (ECH_0039, ECH_0009, and ECH_0230) to be upregulated. Significant changes in the abundance of the outer membrane proteins may be associated with overall changes in the membrane architecture, thereby altering the pathogen’s susceptibility to host defense. The transcriptional changes noted in the ECH_0490 mutant may not have had any negative impact on the pathogen, as the mutant grows similar to the wildtype pathogen both in white-tailed deer (the reservoir host) and in dogs (an incidental host), and in its tick host, *Amblyomma americanum*^5,6^. Transcriptional activity assessment of the genes ECH_0490 (lipoic acid synthetase) and ECH_0492 (putative phosphate ABC transporter), both of which are located up and down stream to the transposon insertion mutation, respectively, suggested that the mutation has no effect on these genes’ transcription (Fig. S4). The diverse changes in the transcriptome of the mutant, while having no impact near the mutation site, suggest that the mutation impacted global gene expression and yet did not adversely affect the pathogen’s survival in vertebrate and tick hosts^5,6^.

The most notable observation was the apparent minimal variation in the transcriptome of the ECH_0660 mutant compared to the wildtype *E*. *chaffeensis*. Importantly, mutation within ECH_0660 gene causes severe growth defects *in vivo* in vertebrate hosts^5,6^. Further, infection with this mutant also initiates a strong host response and confers protection against wildtype pathogen infection challenge^14,60^. In the current study, we observed only minor changes in the gene expression in this mutant compared to wildtype. The minor changes in gene expression included genes encoding for putative nitrogen regulation protein, ABC transporter, heme exporter protein and GroES, but the variations were significantly less compared to numerous changes described in the previous two mutants. Together, these data suggest that the mutation in ECH_0660 gene led to fewer transcriptional alterations. Assuming that the proteomes of the wild type and mutant strains of *E*. *chaffeensis* are similarly altered as the transcriptomes, then ECH_0660 mutant proteome may be very similar to the wildtype bacterium. The greater degree of similarity between this mutant and the wildtype may enable the vertebrate hosts to recognize this mutant as closer to wildtype organism, thus inducing a stronger host response that mimics wildtype infection^14,60^. The replication defect reported earlier with this mutant may have resulted due to the loss of gene expression from fewer genes such as ECH_0659 and ECH_0660, while maintaining most of the transcriptome similar to the wildtype.

## Conclusions

RNA deep sequencing studies in intracellular bacteria are still a major challenge. The RNA seq data reported here provide the first snapshot of comparative transcriptomics of *E*. *chaffeensis*. Sequencing of enriched bacterial RNA from wildtype and mutant strains yielded a high coverage of genes. A mutation in the ORF of ECH_0379 gene caused drastic down-regulation of genes leading to metabolic depression, which may have contributed to the mutant’s attenuation in vertebrate hosts. While a mutation downstream to the protein coding sequence of ECH_0490 gene induced global changes in gene expression, up regulation of stress response regulatory genes may have helped the mutant survive in the vertebrate hosts and tick hosts. A mutation within ECH_0660 gene coding sequence resulted in few transcriptional changes, thus keeping the integrity of its transcriptome similar to wildtype. While the transcriptome data are suggestive of protein expression variations, additional experimental validation from protein analysis studies is necessary to confirm the results. Together, this study offers the first detailed description of transcriptome data for *E*. *chaffeensis*, suggesting that variations observed in the pathogen’s ability to survive in a host and the host’s ability to induce protection against the pathogen may be the result of global changes in the gene expression, which in turn may impact changes in the pathogen’s proteome.

## Materials and Methods

### *In vitro* cultivation and cell-free *E*. *chaffeensis* recovery

*E*. *chaffeensis* Arkansas isolate wildtype and the mutants were grown in the canine macrophage cell line, DH82^58,61^. Isolation and purification of cell-free *E*. *chaffeensis* wildtype and its mutants were carried out as outlined in Fig. S5. Briefly, the bacterial infection rate in DH82 cells was assessed with Diff-Quik staining. After 72 h of infection when the infection reached to about 80–90%, the culture from four T-150 confluent flasks was harvested and centrifuged at 500 × g for 5 min. Cellular pellets were resuspended in 1 × phosphate buffered saline (PBS) containing protease inhibitors (Roche, Indianapolis, IN) and cells were homogenized on ice by passing through, 15–20 strokes with a 23 g needle in a 10 mL syringe. Efficiency of homogenization, 80–90% lysis, was checked under light microscope. Whole cell lysate was centrifuged at 500 × g for 5 min at 4 °C. The resulting supernatant containing cell-free *Ehrlichia* organisms was filtered through a 2 µm sterile membrane filter (Millipore, Billerica, MA). Cell-free *Ehrlichia* from filtrates were pelleted by centrifuging at 15,000 × g for 15 min and the pellet was suspended in PBS and then layered onto 30% diatrizoate meglumine and sodium solution (Renografin) MD-76R (Mallinckrodt Inc, St. Louis, MO). The suspension was centrifuged for 1 h at 100,000 × g at 4 °C in a S50-ST swinging bucket rotor (Beckman, Indianapolis, IN). The pellet of cell-free *Ehrlichia* were washed at 15,000 × g for 15 min and used for experiments.

### Bacterial mRNA enrichment and sequencing

Figure S6 outlines the workflow for bacteria mRNA enrichment and cDNA library preparation and RNA sequencing. Briefly, RNA form wildtype and mutants were isolated from purified cell-free *Ehrlichia* using TRIzol Reagent (Sigma-Aldrich, St. Louis, MO). RNA samples were then treated with DNase I (Invitrogen, Carlsbad, CA) and bacterial RNA was enriched by removing host 18 S rRNA, 28 S rRNA, and polyadenylated mRNA using MICROBEnrich Kit (Ambion, Foster City, CA). The quantity and integrity of bacterial RNA before and after enrichment was assessed using a NanoDrop 2000 spectrophotometer (Thermo Scientific, Waltham, MA) and Agilent 2100 Bioanalyzer (Agilent Technologies, Santa Clara, CA). The Ribo-Zero Magnetic Kit was used to isolate mRNA from total RNA samples and then fragmented into short fragments as per the manufacturer’s protocols (Epicentre, Madison, WI). Subsequently, cDNA was synthesized using the mRNA fragments as templates. Libraries of cDNAs for wildtype and mutants were prepared using the TruSeq RNA Sample Prep Kit (Illumina, Ingolstadt, Germany). Sample libraries were quantified using Agilent 2100 Bioanaylzer and library quality was assessed by Real-Time PCR (ABI StepOnePlus) prior to subjecting the samples to sequencing on Illumina HiSeqTM 4000 (Beijing Genomics Institute (BGI), Philadelphia, PA).

### Bioinformatics analysis

The original image data were transferred into raw sequence data via base calling. Raw reads were subjected to quality assessment to determine whether the raw reads were qualified for mapping (Fig. S5). The bases with low quality (<20) were excluded from the analysis. Raw reads were then filtered to remove adapter sequences and low quality reads, then clean reads were aligned to the *E*. *chaffeensis* Arkansas strain complete genome as per the first annotated GenBank # CP000236.1 using SOAPaligner/SOAP2^62^. We opted to use this accession number because our prior publications, and similarly other investigators, widely used it for referring to gene names and numbers listed in it. Not more than five mismatches were allowed in the alignment, which is a standard cut off used for the alignment analysis. The alignment data were used to calculate distribution of reads on reference genes and determine the gene coverage. Alignment results were assessed for quality check and then proceed with analysis of DGE. The gene expression level was calculated using RPKM method of normalizing for total read length and the number of sequencing reads^63^. We used p-value < 0.05, False Discovery Rate (FDR) ≤ 0.001, and the absolute value of Log2 Ratio ≥ 1 as the threshold to judge the significance difference in gene expression. The FDR uses accurate p-values as a measure of control in multiple sample comparison of RNA seq data. Corrections for false positive and false negative errors were performed using the method described by Benjamini and Yekutieli^64^.

### Quantitative real-time reverse transcription PCR

SYBR green detection-based quantitative real-time reverse transcription PCR (qRT-PCR assays were performed to validate the gene expression changes observed in the RNA seq data analysis. Wildtype, ECH_0379, ECH_0490, and ECH_0660 mutants’ RNAs used in generating the RNA seq data were also used to determine transcript levels by performing quantitative RT-PCR by SYBR Green assays using a SuperScript® III Platinum SYBR Green One-Step qRT-PCR Kit (Invitrogen, Carlsbad, CA). RNA was reverse transcribed from all the replicates using SuperScript III and then quantitative-PCRs were performed in a 25 μL reaction containing 0.5 μM each of forward and reverse primers. Thermal cycler conditions were; 94 °C for 15 sec, 60 °C for 30 sec, and 74 °C for 15 sec for 40 cycles. Thirteen randomly selected differentially transcribed genes were used in validation experiments using StepOnePlus™ Real-Time PCR instrument (Applied Biosystems, Foster City, CA) and the data were analyzed by StepOne Software v2.3. *E*. *chaffeensis* 16 S rRNA was quantitated by real-time RT-PCR as described in^27^ and used for normalization of RNA concentrations among different RNA batches, prior to performing the validation experiments. For qRT-PCR data, the delta-delta Ct (ΔΔCt) calculation was employed to calculate relative change in the expression and fold change was obtained by averaging the replicate values of gene expression and the standard error. Semi-quantitative one-step RT-PCR (Life Technologies, Carlsbad, CA) targeting to *E*. *chaffeensis* genes ECH_0490 and ECH_0492 near the transposon mutation downstream to ECH_0490 gene was performed with 30 cycles of amplification using the gene specific primers as described in a previous study^6^. Briefly, RNA from wildtype and ECH_0490 mutant were used as the templates for RT-PCR. One tube without reverse transcriptase or template RNA was used as negative control. One tube with DNA as the template was used as positive control. Thermal cycler conditions were as follows: 50 °C for 1 h for reverse transcription step then followed by 35 cycles of 94 °C for 30 sec, 55 °C for 30 sec, and 72 °C for 30 sec; finally a 2-min 72 °C extension step was part of the reaction.

## Electronic supplementary material

Supplementary Figures and Tables

## References

[1] Gayle A, Ringdahl E (2001). Tick-borne diseases. Am Fam Physician.

[2] Dawson JE (1996). Human ehrlichiosis in the United States. Curr Clin Top Infect Dis.

[3] Dawson JE, Ewing SA (1992). Susceptibility of dogs to infection with *Ehrlichia chaffeensis*, causative agent of human ehrlichiosis. Am J Vet Res.

[4] Dumler JS, Bakken JS (1995). *Ehrlichial* diseases of humans: emerging tick-borne infections. Clin Infect Dis.

[5] Cheng C (2013). Targeted and random mutagenesis of *Ehrlichia chaffeensis* for the identification of genes required for *in vivo* infection. PLoS Pathog.

[6] Cheng C, Nair AD, Jaworski D, Ganta RR (2015). Mutations in *Ehrlichia chaffeensis* causing polar effects in gene expression and differential host specificities. PLoS One.

[7] Moumène A, Meyer DF (2016). *Ehrlichia’s* molecular tricks to manipulate their host cells. Microbes Infect.

[8] Kuriakose JA, Miyashiro S, Luo T, Zhu B, McBride JW (2011). *Ehrlichia chaffeensis* transcriptome in mammalian and arthropod hosts reveals differential gene expression and post transcriptional regulation. PLoS One.

[9] Unver A, Rikihisa Y, Stich RW, Ohashi N, Felek S (2002). The omp-1 major outer membrane multigene family of *Ehrlichia chaffeensis* is differentially expressed in canine and tick hosts. Infect Immun.

[10] Liu H, Von Ohlen T, Cheng C, Faburay B, Ganta RR (2013). Transcription of *Ehrlichia chaffeensis* genes is accomplished by RNA polymerase holoenzyme containing either sigma 32 or sigma 70. PLoS One.

[11] Kuczynska-Wisnik D., Cheng, C., Ganta, R.R. & Zolkiewski, M. Protein aggregation in *Ehrlichia chaffeensis* during infection of mammalian cells. *FEMS Microbiol Lett*. **364** (2017). 10.1093/femsle/fnx059 PMC5399918 28333306

[12] Hammac GK, Pierlé SA, Cheng X, Scoles GA, Brayton KA (2014). Global transcriptional analysis reveals surface remodeling of *Anaplasma marginale* in the tick vector. Parasit Vectors..

[13] McClure EE (2017). Engineering of obligate intracellular bacteria: progress, challenges and paradigms. Nat Rev Microbiol.

[14] Nair AD (2015). Attenuated mutants of *Ehrlichia chaffeensis* induce protection against wild-type infection challenge in the reservoir host and in an incidental host. Infect. Immun.

[15] Pierle SA, Dark MJ, Dahmen D, Palmer GH, Brayton KA (2012). Comparative genomics and transcriptomics of trait-gene association. BMC Genomics.

[16] Albrecht M, Sharma CM, Reinhardt R, Vogel J, Rudel T (2010). Deep sequencing-based discovery of the *Chlamydia trachomatis* transcriptome. Nucleic Acids Res.

[17] Martin J, Zhu W, Passalacqua KD, Bergman N, Borodovsky M (2010). *Bacillus anthracis* genome organization in light of whole transcriptome sequencing. BMC Bioinforma.

[18] Dunning Hotopp JC (2006). Comparative Genomics of Emerging Human Ehrlichiosis Agents. PLoS Genet..

[19] Collins NE (2005). The genome of the heartwater agent *Ehrlichia ruminantium* contains multiple tandem repeats of actively variable copy number. Proc Natl Acad Sci USA.

[20] Frutos R (2006). Comparative genomic analysis of three strains of *Ehrlichia ruminantium* reveals an active process of genome size plasticity. J Bacteriol.

[21] Luo T, McBride JW (2012). *Ehrlichia chaffeensis* TRP32 interacts with host cell targets that influence intracellular survival. Infect Immun..

[22] Wakeel A, den Dulk-Ras A, Hooykaas PJ, McBride JW (2011). Ehrlichia chaffeensis tandem repeat proteins and ank200 are type 1 secretion system substrates related to the repeats-in-toxin exoprotein family. Front Cell Infect Microbiol.

[23] Zhu B (2009). Nuclear translocated *Ehrlichia chaffeensis* ankyrin protein interacts with a specific adenine-rich motif of host promoter and intronic Alu elements. Infect Immun.

[24] Noroy C, Meyer DF (2017). Comparative genomics of the zoonotic pathogen *Ehrlichia chaffeensis* reveals candidate type IV effectors and putative host cell targets. Front Cell Infect Microbiol..

[25] Westermann AJ, Gorski SA, Vogel J (2012). Dual RNA-seq of pathogen and host. Nat. Rev. Microbiol..

[26] Kumar N (2016). Efficient Enrichment of Bacterial mRNA from Host-Bacteria Total RNA Samples. Sci Rep.

[27] Sirigireddy KR, Ganta RR (2005). Multiplex detection of *Ehrlichia* and *Anaplasma* species pathogens in peripheral blood by real-time reverse transcriptase-polymerase chain reaction. J Mol Diagn.

[28] Westermann AJ, Barquist L, Vogel J (2017). Resolving host-pathogen interactions by dual RNA-seq. PLoS Pathog.

[29] Schroeder CL (2016). Identification and characterization of novel small RNAs in *Rickettsia prowazekii*. Front Microbiol.

[30] Schroeder CL (2015). Bacterial small RNAs in the Genus Rickettsia. BMC Genomics.

[31] Ohashi N, Zhi N, Zhang Y, Rikihisa Y (1998). Immunodominant major outer membrane proteins of *Ehrlichia chaffeensis* are encoded by a polymorphic multigene family. Infect Immun.

[32] Reddy GR (1998). Molecular characterization of a 28 kDa surface antigen gene family of the tribe *Ehrlichia*. Biochem Biophys Res Commun.

[33] Yu XJ, McBride JW, Zhang X, Walker DH (2000). Characterization of the complete transcriptionally active *Ehrlichia chaffeensis* 28 kDa outer membrane protein multigene family. Gene.

[34] Lin M, Rikihisa Y (2007). Degradation of p22phox and inhibition of superoxide generation by *Ehrlichia chaffeensis* in human monocytes. Cell Microbiol.

[35] Green, E. R. & Mecsas, J. Bacterial secretion systems: An Overview. *Microbiol Spectr* **4** (2016) 10.1128/microbiolspec.VMBF-0012-2015 PMC4804464 26999395

[36] Rapisarda C, Fronzes R (2017). Secretion systems used by bacteria to subvert host functions. Curr Issues Mol Biol..

[37] Rikihisa Y, Lin M, Niu H, Cheng Z (2009). Type IV secretion system of *Anaplasma phagocytophilum* and *Ehrlichia chaffeensis*. Ann N Y Acad Sci.

[38] Lopez JE (2007). Immunogenicity of *Anaplasma marginale* type IV secretion system proteins in a protective outer membrane vaccine. Infect Immun.

[39] Felek S, Huang H, Rikihisa Y (2003). Sequence and expression analysis of virB9 of the type IV secretion system of *Ehrlichia canis* strains in ticks, dogs, and cultured cells. Infect Immun.

[40] Sexton JA, Vogel JP (2002). Type IVB secretion by intracellular pathogens. Traffic.

[41] Ohashi N, Zhi N, Lin Q, Rikihisa Y (2002). Characterization and transcriptional analysis of gene clusters for a type IV secretion machinery in human granulocytic and monocytic ehrlichiosis agents. Infect. Immun..

[42] Pruneau L (2014). Understanding Anaplasmataceae pathogenesis using “Omics” approaches. Front Cell Infect Microbiol.

[43] Lin M, Kikuchi T, Brewer HM, Norbeck AD, Rikihisa Y (2011). Global proteomic analysis of two tickborne emerging zoonotic agents: *anaplasma phagocytophilum* and *ehrlichia chaffeensis*. Front Microbiol.

[44] Mosavi LK, Cammett TJ, Desrosiers DC, Peng ZY (2004). The ankyrin repeat as molecular architecture for protein recognition. Protein Sci..

[45] Patiño-Ruiz M, Ganea C, Fendler K, Călinescu O (2017). Competition is the basis of the transport mechanism of the NhaB Na+/H+ exchanger from *Klebsiella pneumoniae*. PLoS One..

[46] Krulwich TA, Hicks DB, Ito M (2009). Cation/proton antiporter complements of bacteria: why so large and diverse?. Mol Microbiol.

[47] Murphy TF, Brauer AL, Johnson A, Kirkham C (2016). ATP-binding cassette (ABC) transporters of the human respiratory tract pathogen, *Moraxella catarrhalis*: role in virulence. PLoS One.

[48] Tjale MA, Pretorius A, Josemans A, Kleef MV, Liebenberg J (2018). Transcriptomic analysis of *Ehrlichia ruminantium* during the developmental stages in bovine and tick cell culture. Ticks Tick Borne Dis..

[49] Perez Vidakovics ML, Riesbeck K (2009). Virulence mechanisms of Moraxella in the pathogenesis of infection. Curr Opin Infect Dis.

[50] Eisenreich W, Heesemann J, Rudel T, Goebel W (2013). Metabolic host responses to infection by intracellular bacterial pathogens. Front Cell Infect Microbiol.

[51] Olive AJ, Sassetti CM (2016). Metabolic crosstalk between host and pathogen: sensing, adapting and competing. Nat Rev Microbiol.

[52] Sinclair SH, Rennoll-Bankert KE, Dumler JS (2014). Effector bottleneck: microbial reprogramming of parasitized host cell transcription by epigenetic remodeling of chromatin structure. Front Genet.

[53] Gillespie JJ (2010). Phylogenomics reveals a diverse Rickettsiales type IV secretion system. Infect Immun.

[54] Zhang T (2013). Aggregate-reactivation activity of the molecular chaperone ClpB from *Ehrlichia chaffeensis*. PLoS One..

[55] Zolkiewski M, Zhang T, Nagy M (2012). Aggregate reactivation mediated by the Hsp100 chaperones. Arch Biochem Biophys.

[56] Kumagai Y, Huang H, Rikihisa Y (2006). Expression and porin activity of P28 and OMP-1F during intracellular *Ehrlichia chaffeensis* development. J Bacteriol.

[57] Marcelino I (2015). Comparative proteomic profiling of *Ehrlichia ruminantium* pathogenic strain and its high-passaged attenuated strain reveals virulence and attenuation-associated proteins. PLoS One..

[58] Singu, V. *et al*. Unique macrophage and tick cell-specific protein expression from the p28/p30 Omp multigene locus in *Ehrlichia* species. *Cell Microbiol*. **8**, 1475–1487 (2006). 10.1111/j.1462-5822.2006.00727.x 16922866

[59] Seo GM, Cheng C, Tomich J, Ganta RR (2008). Total, membrane, and immunogenic proteomes of macrophage- and tick cell-derived *Ehrlichia chaffeensis* evaluated by LC-MS/MS and MALDI-TOF methods. Infect Immun.

[60] McGill JL (2016). Vaccination with an attenuated mutant of *Ehrlichia chaffeensis* induces pathogen-specific CD4 + T cell immunity and protection from tick-transmitted wild-type challenge in the canine host. PLoS One.

[61] Cheng, C. & Ganta, R. R. Laboratory maintenance of *Ehrlichia chaffeensis* and *Ehrlichia canis* and recovery of organisms for molecular biology and proteomics studies. *Curr Protoc Microbiol* **3**. Chapter 3A (2008) 10.1002/9780471729259.mc03a01s9 PMC8336927 18770537

[62] Li R (2009). SOAP2: An improved ultrafast tool for short read alignment. Bioinformatics.

[63] Mortazavi A, Williams BA, McCue K, Schaeffer L, Wold B (2008). Mapping and quantifying mammalian transcriptomes by RNA-Seq. Nature Med.

[64] Benjamini Y, Yekutieli D (2001). The control of the false discovery rate in multiple testing under dependency. The Annals of Statistics.

