# Supplementary material for: Impact of Three Different Mutations in Ehrlichia chaffeensis in Altering the Global Gene Expression Patterns
Source: Sci Rep. 2018 Apr 18;8:6162. doi: 10.1038/s41598-018-24471-3 (PMC5906474; doi:10.1038/s41598-018-24471-3)

Impact of Three Different Mutations *in Ehrlichia chaffeensis in Altering the Global* Gene Expression Patterns

Chandramouli Kondethimmanahalli and Roman Ganta*

Center of Excellence for Vector-Borne Diseases, Department of Diagnostic Medicine/Pathobiology, College of Veterinary Medicine, Kansas State University, Manhattan, Kansas, USA 66506

*Corresponding author; rganta@vet.k-state.edu

Fig. S1. Bioanalyzer analysis data of cell-free *E. chaffeensis* RNA before (A) and after RNA enrichment (B) steps. Lane L, RNA ladder; 1, Wildtype ECH_7000; 2, mutant ECH_0660; 3, mutant ECH_0379; 4, mutant ECH_0490. Note: The Bioanalyzer data for lane 4 of panel B was from a different gel ran separately and RNA markers to define the sizes of the resolved RNAs were also ran separately. The original data files for lane 4 and its associated markers were presented on the last page of this file (page # 41).

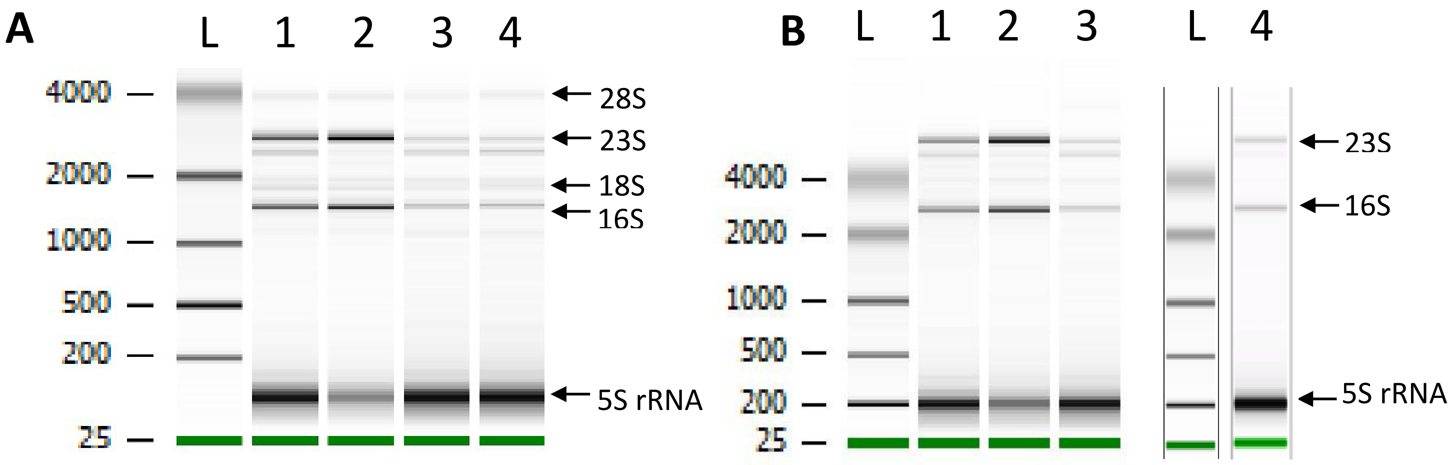

Fig. S2. Chromatogram of cell-free *E. chaffeensis.* RNA before (A) and after enrichment (B). 18S and 28S peaks represent host cell ribosomal RNAs; 16S and 23S represent *E. chaffeensis* ribosomal RNAs.

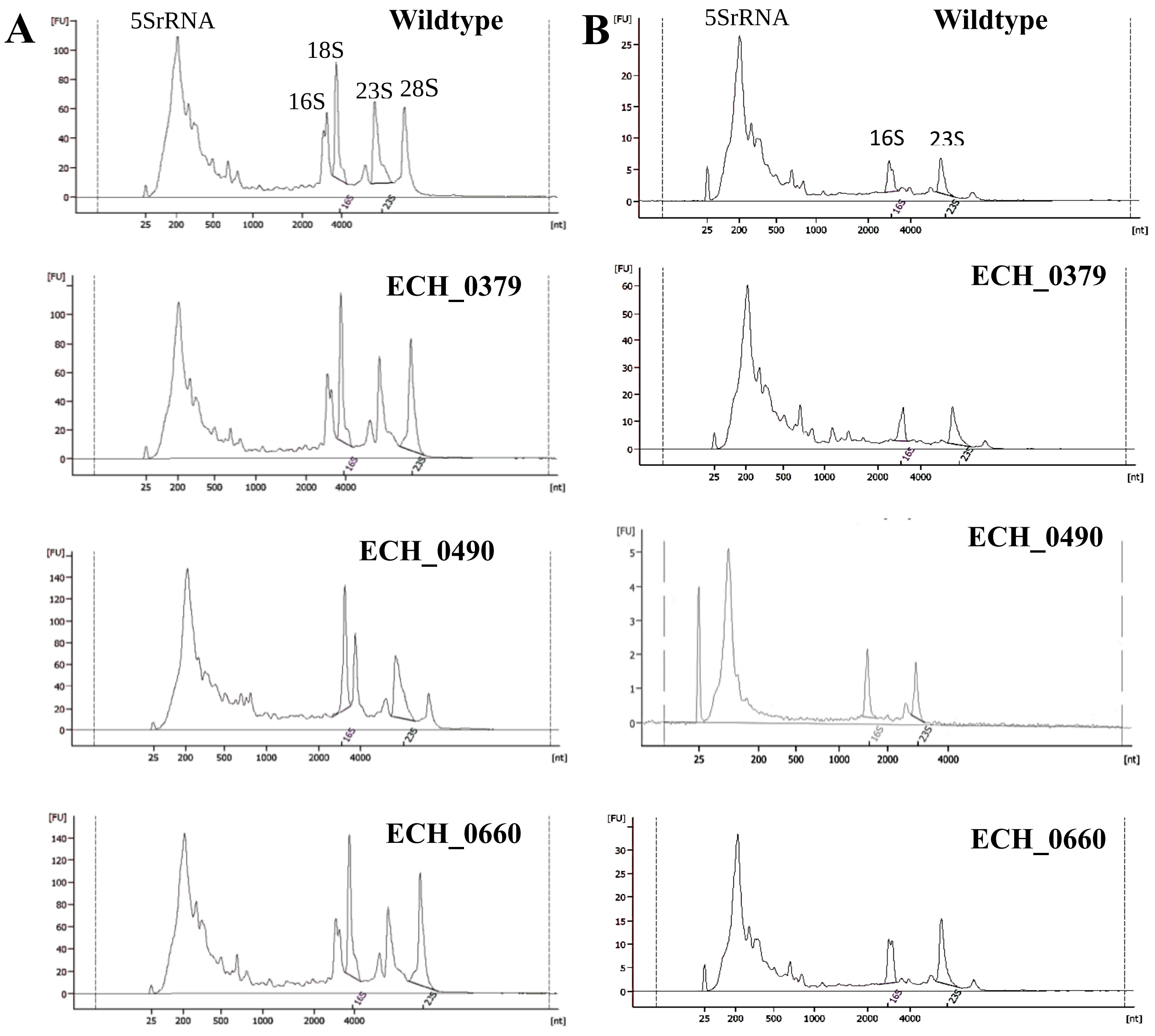

Fig. S3. Distribution of differentially expressed genes in mutants ECH_0379 (A) and ECH_0490 (B) compared to wildtype. Transcripts with ≥1 FPKM and minimum of 10 mapped reads were used. The plot is on a log-transformed scale.

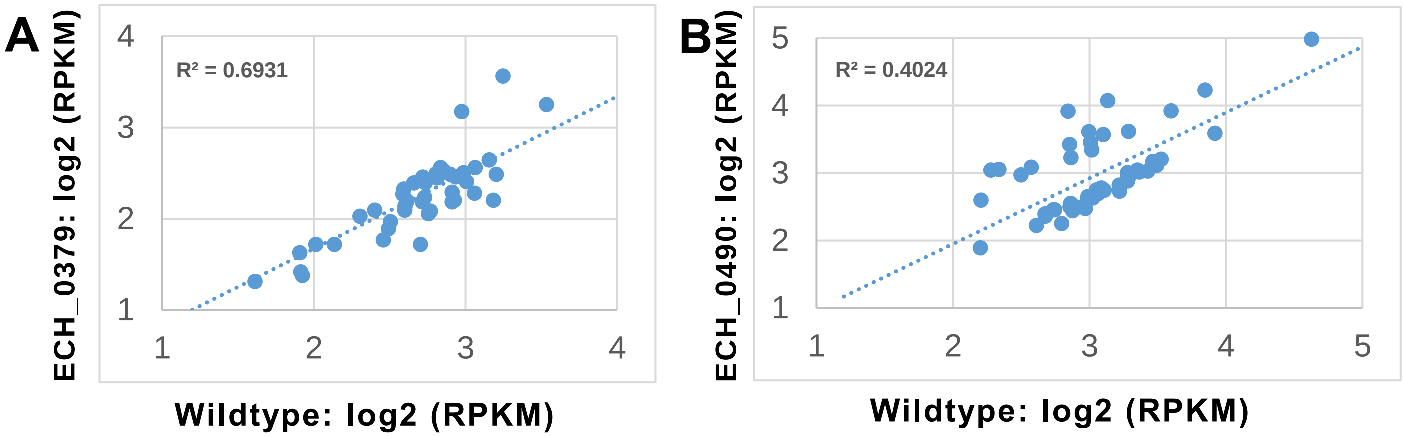

Fig. S4. Semi-quantitative RT-PCR targeting to *E. chaffeensis* genes ECH_0490 and ECH_0492 in the ECH_0490 mutant. RNA from wild-type and mutant was used as templates for RT-PCR analysis. Lane 1 and 6, RNA from wild-type *E.chaffeensis*; lane 2 and 7 RNA from the mutant; lane 3 and 8 without reverse transcriptase; lane 4 and 9 without a template; lane 5 and 10 genomic DNA (positive control).

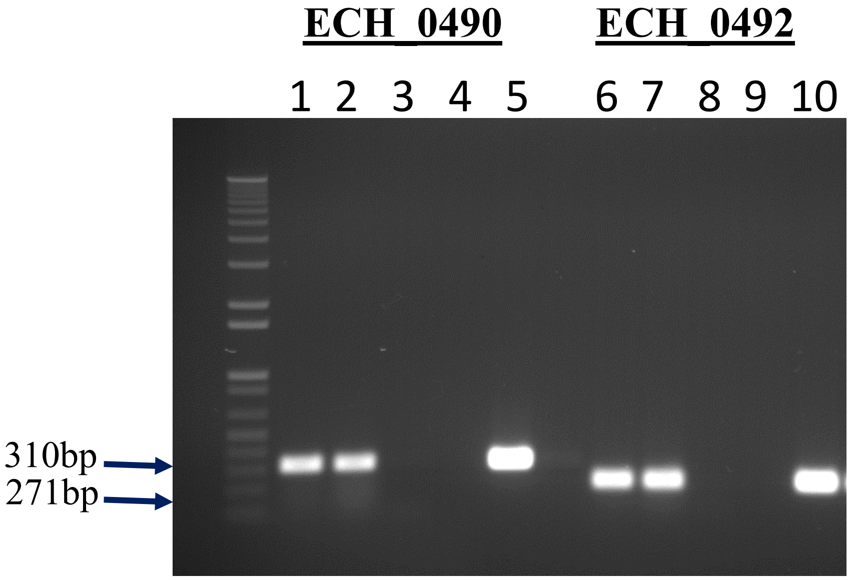

Fig. S5. Schematic diagram of infection, isolation and purification of cell-free *E. chaffeensis*.

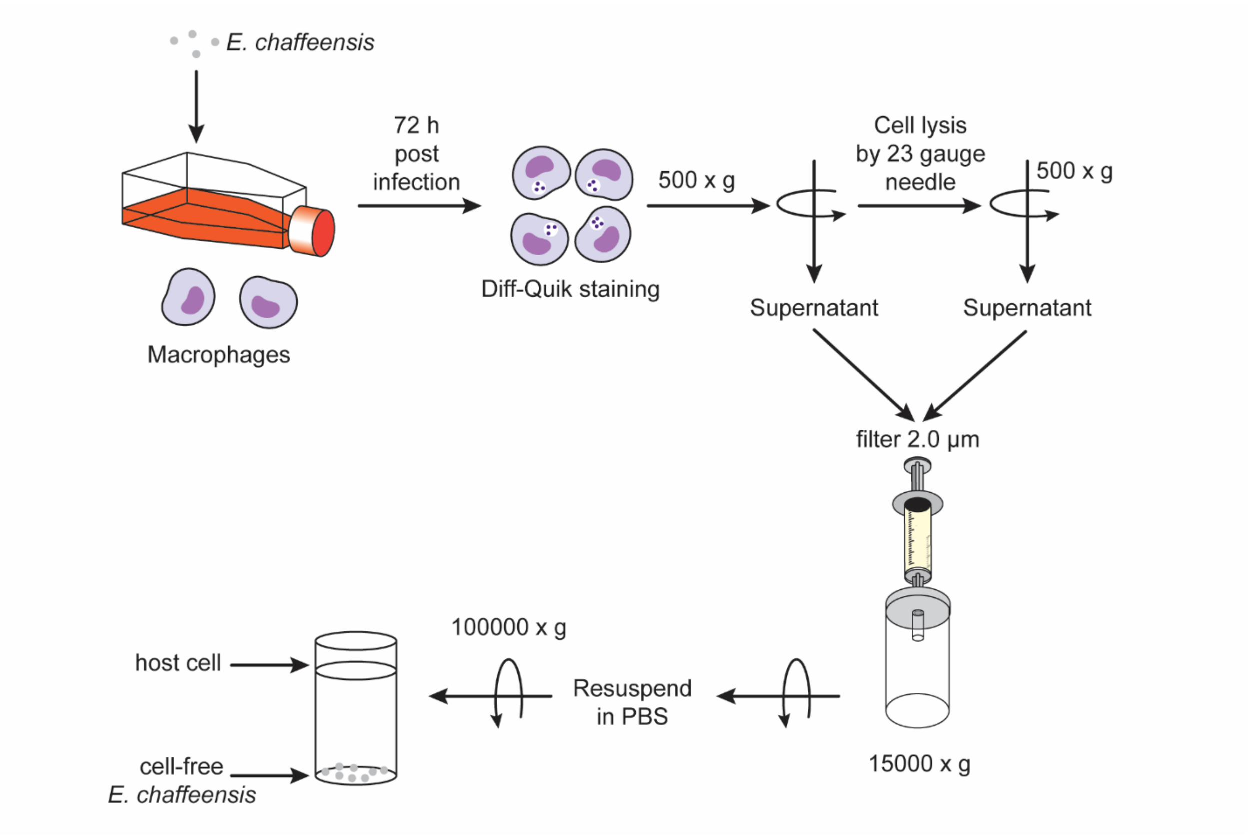

Note: The above illustration was made by Ms. Mal Rooks Hoover, Kansas State University. We thank Ms. Hoover for her help in preparing this schematic diagram as per our direction. She granted the permission in writing for its publication.

Fig. S6. Workflow of sample preparation and RNA sequencing of *E. chaffeensis* wildtype and mutants organisms.

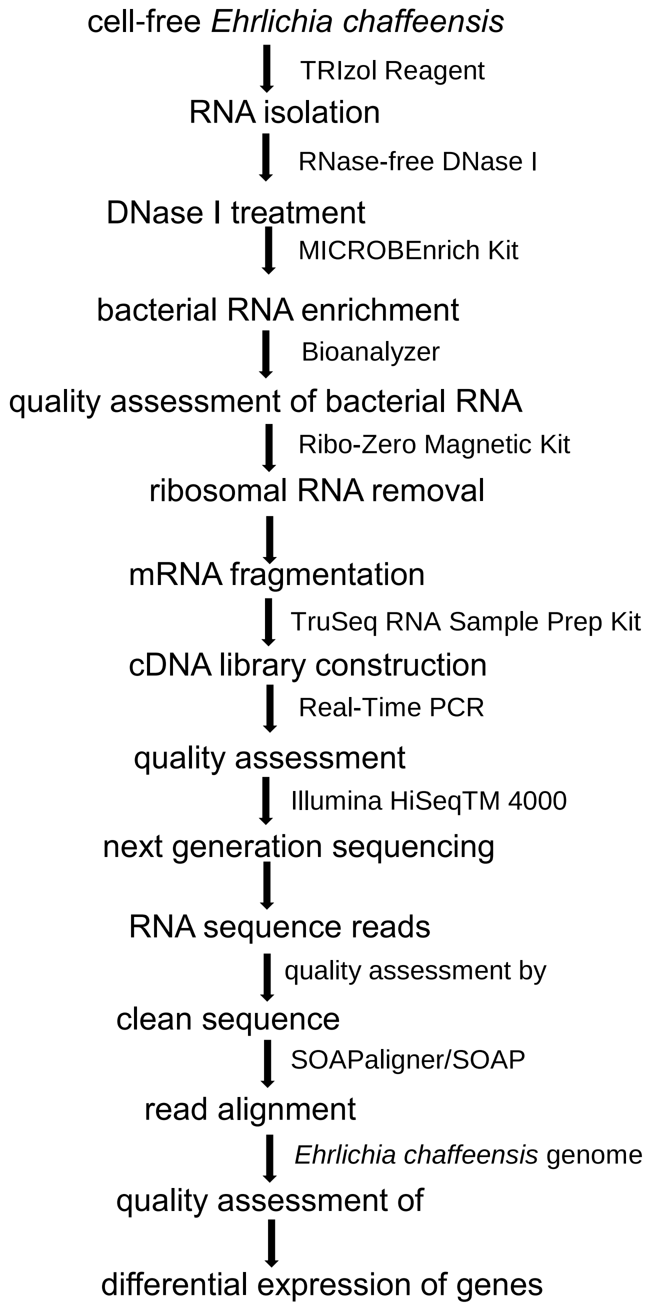

Table S1: *E. chaffeensis* genes identified in wildtype and mutants by mapping the reads against the reference genome of *E. chaffeensis* Arkansas (GenBank # CP000236.1). Blank cells in table indicate that the expression values are not determined.					
Gene ID	Product	Wildtype RPKM	ECH_0379 RPKM	ECH_0490 RPKM	ECH_0660 RPKM
ECH_0001	chromosome partitioning protein, ParB family;	66.7072	52.645	267.662	66.136
ECH_0002	16S rRNA processing protein RimM;	267.208	195.06	232.814	211.93
ECH_0003	tRNA (guanine-N1)-methyltransferase;	2602.4	2718.1	2247.97	3681.1
ECH_0004	ribosomal protein L19;	3780.85	3080.6	2242.17	3986.5
ECH_0005	competence/damage-inducible protein CinA C-terminal domain;	276.177	196.65	212.997	248.46
ECH_0006	threonyl-tRNA synthetase;	1004	1114.2	1023.87	887.85
ECH_0007	translation initiation factor IF-3;	2214.83	1950	2032.65	1806.6
ECH_0008	P-loop hydrolase family protein;	2879.4	1697.1	5032.04	2216
ECH_0009	putative membrane protein;	7047.64	8575.6	16828.8	9129.3
ECH_0010	hypothetical protein;	3668.83	2730.9	5511.99	3101.1
ECH_0011	glyceraldehyde-3-phosphate dehydrogenase, type I;	324.57	376.49	641.842	299.75
ECH_0012	Es1 family protein;	472.919	473.69	524.576	620.9
ECH_0013	putative pyrroline-5-carboxylate reductase;	716.491	604.84	726.851	708.2
ECH_0014	DNA polymerase III, subunits gamma and tau;	715.401	547.07	554.065	538.21
ECH_0015	conserved hypothetical protein;	1545.19	1037	1395.44	1392.2
ECH_0016	aspartate-semialdehyde dehydrogenase;	501.628	659.88	505.551	724.87
ECH_0018	S-adenosylmethionine synthetase;	377.755	364.38	548.751	429.23
ECH_0020	ubiquinone biosynthesis hydroxylase, UbiH/UbiF/VisC/COQ6 family;	170.339	125.77	275.779	109.42
ECH_0021	conserved hypothetical protein;	397.115	210.65	396.346	291.96
ECH_0023	glycyl-tRNA synthetase, alpha subunit;	1801.17	1749	1358.42	1886.5
ECH_0024	glycyl-tRNA synthetase, beta subunit;	561.858	386.62	606.541	378.99
ECH_0025	chaperone protein DnaJ;	429.018	575.56	741.505	481.42
ECH_0026	nicotinate-nucleotide pyrophosphorylase;	433.97	437.43	469.829	339.1
ECH_0027	putative membrane protein, TIGR00023;	134.886	238.28	792.217	214.57
ECH_0028	crossover junction endodeoxyribonuclease RuvC;	516.513	508.26	370.879	443.19
ECH_0029	cytochrome c oxidase, subunit III;	2155.69	2165	2164.43	1668.3
ECH_0030	uroporphyrinogen decarboxylase;	228.408	253.9	579.382	271.79
ECH_0031	hemolysin;	470.629	523.5	590.148	521.99

ECH_0032	phage prohead protease, HK97 family;	82.835	26.064		58.14
ECH_0033	phage portal protein, HK97 family;	41.4532	20.41		26.993
ECH_0035	putative biotin synthesis protein BioC;	407.75	242.48	353.681	267.49
ECH_0036	quinolinate synthetase complex, subunit A;	756.976	714.81	643.554	807.23
ECH_0038	ferredoxin A;	112.982	166.92	393.663	192.23
ECH_0039	120 kDa immunodominant surface protein;	316.256	495.77	931.81	413.87
ECH_0040	type IV secretion system protein VirD4;	825.555	901.06	603.242	730.54
ECH_0041	type IV secretion system protein VirB11;	783.237	830.35	453.337	734.3
ECH_0042	type IV secretion system protein VirB10;	1221.68	1161.9	852.046	1130.3
ECH_0043	type IV secretion system protein VirB9;	850.763	845.47	480.117	747.21
ECH_0044	type IV secretion system protein VirB8;	1733.53	1250.2	434.943	1320.4
ECH_0045	putative GTP cyclohydrolase II;	384.785	449.57	718.863	439.12
ECH_0048	TPR domain protein;	422.257	184.54	229.553	178.25
ECH_0049	hypothetical protein;	1294.48	397.03	581.209	440.38
ECH_0050	diaminopimelate epimerase;	401.232	473.17	418.443	430.77
ECH_0052	conserved hypothetical protein;	457.936	164.52		149.45
ECH_0055	phosphoglycerate kinase;	443.541	349.56	359.432	353.74
ECH_0056	putative exodeoxyribonuclease VII, large subunit;	91.4889	120.85	127.51	93.511
ECH_0057	zinc finger-like domain protein;	172.908	156.04	563.433	236.06
ECH_0058	2,3,4,5-tetrahydropyridine-2-carboxylate N-succinyltransferase;	1902.02	2742.8	1006.7	3089.7
ECH_0060	tRNA modification GTPase TrmE;	179.101	175.03	279.376	210.23
ECH_0061	putative flavoprotein;	199.434	165.75	238.297	199.27
ECH_0062	ATP-dependent DNA helicase RecG;	78.5055	73.684	1006.889	83.673
ECH_0063	putative NADH-ubiquinone oxidoreductase, homolog;	224.907	196.56	705.427	223.96
ECH_0064	DnaK suppressor protein;	601.46	756.05	479.242	674.97
ECH_0065	putative heme exporter protein CcmB;	235.804	193.13	253.716	210.93
ECH_0067	cation diffusion facilitator transporter family protein;	593.705	490.64	702.472	500.2
ECH_0069	conserved hypothetical protein;	180.367	66.277		67.84
ECH_0071	ribosomal protein S20;	6265.18	5151.6	4807.4	7407.4
ECH_0072	1-acyl-sn-glycerol-3-phosphate acyltransferase family protein;	127.641	180.09		164.54
ECH_0073	peptide deformylase;	325.494	451.66	969.895	392.5
ECH_0074	uracil-DNA glycosylase, family 4;	576.335	737.82	625.576	821.35

ECH_0076	putative DNA replication and repair protein RecF;	287.159	59.972	213.924	56.14
ECH_0077	ornithine carbamoyltransferase;	1405.67	1138.5	1418.81	727.57
ECH_0078	hypothetical protein;	1538.63	927.88	1929.58	893.51
ECH_0079	hypothetical protein;	8953.79	10651	12156.8	8293.1
ECH_0080	DNA polymerase I;	486.359	391.44	479.242	409.91
ECH_0082	ribulose-phosphate 3-epimerase;	167.622	197.19	395.532	184.52
ECH_0084	conserved hypothetical protein;	527.759	440.41	812.628	365.63
ECH_0085	ABC transporter, ATP-binding protein;	1119.2	438.7	523.357	292.15
ECH_0088	octaprenyl-diphosphate synthase;	193.386	98.67	205.075	138.8
ECH_0089	glutamine synthetase, type I;	2144.31	2090.8	1694.14	1956.7
ECH_0091	tyrosyl-tRNA synthetase;	912.481	932.31	844.107	720.75
ECH_0092	5-aminolevulinic acid synthase;	1046.55	1116.4	523.311	1087.8
ECH_0093	conserved hypothetical protein;	107.47	67.893		60.866
ECH_0095	protein-export membrane protein SecF;	1921.25	551.26	990.271	640.13
ECH_0097	fructose-biphosphate aldolase, class I;	2049.45	2926.1	1804.21	2783.3
ECH_0098	pyruvate dehydrogenase complex, E2 component, dihydrolipoamide acetyltransferase;	1130.83	1430.8	972.276	1588.4
ECH_0105	hypothetical protein;	147.605	932.86	1182.65	816.13
ECH_0106	hypothetical protein;	44.1482	51.982	682.618	55.079
ECH_0108	hypothetical protein;	310.22	331.76	990.23	218.03
ECH_0113	hypothetical protein;	18.2492	29.215	545.938	22.296
ECH_0115	hypothetical protein;	124.612	92.484	422.861	69.727
ECH_0116	hypothetical protein;	35.1345	51.13		44.993
ECH_0117	hypothetical protein;	98.9284	109.7	541.803	74.41
ECH_0119	hypothetical protein;	33.4694	72.718		24.987
ECH_0120	hypothetical protein;	198.378	239.92	997.675	152.08
ECH_0121	hypothetical protein;	81.9805	76.275	286.376	93.149
ECH_0122	hypothetical protein;	5514.52	5847.3	7539.57	4736.4
ECH_0123	GMP synthase;	435.097	511.18	622.015	529.04
ECH_0124	citrate synthase I;	165.815	169.11	527.511	217.23
ECH_0125	glutamate--cysteine ligase;	385.471	185.12	577.19	268.62
ECH_0126	conserved hypothetical protein;	61.4653	55.396		59.616
ECH_0127	lipoprotein releasing system transmembrane protein, LolC/E family;	627.171	585.47	703.122	586.53
ECH_0128	putative lipoprotein;	712.569	500.36	416.33	488.5

ECH_0129	HemY domain protein;	520.609	427.97	460.279	415.83
ECH_0130	conserved hypothetical protein;	173.46	269.27		274.33
ECH_0131	ATP synthase F1, delta subunit;	1871.03	1691.1	2168.18	1768.4
ECH_0132	ATP synthase F1, alpha subunit;	1248.11	1602.3	1099.44	1621.9
ECH_0133	transcription elongation factor GreA;	335.306	571.15	383.688	577.09
ECH_0134	3,4-dihydroxy-2-butanone 4-phosphate synthase;	550.385	938.09	620.528	911.41
ECH_0135	SsrA-binding protein;	350.808	408.27	345.054	537.71
ECH_0136	valyl-tRNA synthetase;	223.729	227.86	303.895	235.62
ECH_0137	cytochrome c-type biogenesis protein CcmF;	330.156	230.29	457.357	207.99
ECH_0138	aminomethyl transferase family protein;	268.787	196.22	371.026	209.93
ECH_0139	amidophosphoribosyltransferase;	461.748	538.42	363.313	347.74
ECH_0141	peptidyl-tRNA hydrolase;	342.351	449.17	520.183	802.76
ECH_0142	ribosomal 5S rRNA E-loop binding protein Ctc/L25/TL5;	2875.81	2343.9	2146.37	2856.3
ECH_0143	putative competence protein F;	155.166	190.13	270.741	201.03
ECH_0144	succinyl-diaminopimelate desuccinylase;	1837.35	1974.8	1275.89	1666.1
ECH_0146	putative glutathione-regulated potassium-efflux system protein KefB;	1877.75	1910.7	1269.7	1515.6
ECH_0147	conserved hypothetical protein;	4121.06	3681.2	2512.32	2933.3
ECH_0148	putative membrane protein;	466.341	351.95	416.445	514.19
ECH_0149	putative pyruvate dehydrogenase complex, E1 component, beta subunit;	567.198	534.06	498.671	611.97
ECH_0150	hypothetical protein;	846.48	869.97	2009.19	752.55
ECH_0152	tldD protein;	516.565	606.03	1184.28	715.62
ECH_0154	GTP-binding protein YchF;	1151.99	1078.7	962.445	1045.2
ECH_0156	2C-methyl-D-erythritol 2,4-cyclodiphosphate synthase;	70.1265	158.2	396.614	226.14
ECH_0157	2-C-methyl-D-erythritol 4-phosphate cytidylyltransferase;	39.6865	56.116	90.9954	54.63
ECH_0158	hypothetical protein;	113.558	192.17	562.376	172.61
ECH_0159	hypothetical protein;	1307.07	1741.9	2135.36	1155.4
ECH_0160	phosphoribosylaminoimidazole carboxylase, catalytic subunit;	175.525	312.97	500.637	251.78
ECH_0161	hypothetical protein;	81.021	42.253		56.913
ECH_0162	integration host factor, alpha subunit;	817.997	1059.6	469.752	1151.3
ECH_0163	transcriptional regulator, MerR family;	708.581	937.62	713.203	807.77
ECH_0166	conserved hypothetical protein;	42488.4	53185	96364.3	44584

ECH_0167	tryptophanyl-tRNA synthetase;	718.03	839.9	2653.77	643.04
ECH_0168	co-chaperone GrpE;	1332.1	1525.2	1036.01	1658.5
ECH_0169	riboflavin biosynthesis protein RibD;	161.402	130.23	397.149	152.18
ECH_0170	variable length PCR target protein;	17918.5	15320	10436.2	17080
ECH_0171	CTP synthase;	1681.69	1740.9	1149.1	1762.9
ECH_0172	preprotein translocase, SecG subunit;	5505.56	5026.7	4235.47	6092.2
ECH_0173	tyrosine recombinase XerD;	246.008	282.35	250.443	313.1
ECH_0174	ABC transporter, ATP-binding protein;	169.113	161.2	173.624	212.91
ECH_0175	malate dehydrogenase;	2711.22	2998	1326.91	2763
ECH_0176	conserved hypothetical protein;	380.303	357.04	455.307	384.92
ECH_0178	apolipoprotein N-acyltransferase;	729.203	689.44	506.928	634.78
ECH_0179	putative NADH dehydrogenase I, N subunit;	320.861	286.03	495.314	303.35
ECH_0181	hypothetical protein;	2006.78	1525.6	2820.16	1637.9
ECH_0183	mce-related protein;	659.247	628.89	517.582	534.77
ECH_0184	NADH:ubiquinone oxidoreductase family protein;	874.166	997.31	968.265	1039.8
ECH_0185	ATP cone domain protein;	300.428	134.56		142.41
ECH_0187	hypothetical protein;	29.2971	24.678	267.662	24.284
ECH_0188	putative surface protein;	586.722	121.881	244.584	94.135
ECH_0189	putative iron-binding protein;	2044.66	1952	1379.72	2229.9
ECH_0192	ribosomal protein S16;	3521.36	3033.2	3430.94	4111.3
ECH_0193	major facilitator family transporter;	3894.26	2815.8	3053.89	2934.1
ECH_0195	oxidoreductase, short chain dehydrogenase/reductase family;	172.029	170.64	436.895	173.95
ECH_0196	phenylalanyl-tRNA synthetase, alpha subunit;	813.174	1013.4	944.673	149.29
ECH_0197	ribosomal protein L20;	4187.32	4374.6	3605.82	4468.7
ECH_0198	ribosomal protein L35;	4522.63	5973.5	3959.11	5216.6
ECH_0199	conserved hypothetical protein;	5628.22	4658.2	3567.44	4034
ECH_0201	dnaJ domain protein;	428.977	502.6	754.807	464.05
ECH_0202	NifU domain protein;	698.733	681.43	657.356	843.32
ECH_0203	hypothetical protein;	23.0611	32.626		27.826
ECH_0204	thiamine biosynthesis protein ThiS;	149.739	114.4		129.1
ECH_0206	thiamin biosynthesis ThiG;	151.941	234.64	396.614	220.53
ECH_0207	conserved domain protein;	637.675	82.128	292.419	177.65
ECH_0208	glutamyl-tRNA synthetase;	313.18	399.85	330.289	334.8
ECH_0210	surA domain protein;	882.026	802.91	1034.73	969.12

ECH_0211	putative methyltransferase;	407.562	401.74	1172.06	532.89
ECH_0212	putative transporter;	145.024	218.03	480.814	199.33
ECH_0213	putative oxidoreductase;	631.89	1134.1	883.175	1507.8
ECH_0214	putative exodeoxyribonuclease VII, small subunit;	451.926	471.3	581.937	514.48
ECH_0217	tRNA modification enzyme, MiaB family;	186.502	210.44	741.491	194.76
ECH_0218	thioredoxin;	1254.57	1105.4	838.674	1342.8
ECH_0219	type IV secretion system protein, VirB9;	1629.37	1678.9	636.632	1663.3
ECH_0220	pyruvate dehydrogenase complex, E1 component, pyruvate dehydrogenase alpha subunit;	242.583	265.92	144.649	316.53
ECH_0221	putative hydrolase;	128.442	117.21	83.2368	132.58
ECH_0224	inosine-5'-monophosphate dehydrogenase;	726.534	925.97	807.612	894.18
ECH_0225	putative ATP-NAD kinase;	681.741	745.11	1004.77	781.35
ECH_0226	ribosomal protein L31;	2234.36	2246.4	2610.61	2774.1
ECH_0227	malonyl CoA-acyl carrier protein transacylase;	11175.8	10093	10403.5	9370.7
ECH_0229	thymidylate kinase;	1341.17	969.01	2039.73	666.76
ECH_0230	putative membrane protein;	991.5	2096	4109.04	1271.2
ECH_0232	tim44-like domain protein;	488.128	368.83	526.491	345.9
ECH_0233	protein-export protein SecB;	4262.32	2778.4	1715.47	4184.4
ECH_0234	conserved hypothetical protein;	521.075	269.51	384.786	426.86
ECH_0235	peptidase, M16 family;	280.714	261.65	301.514	298.98
ECH_0237	conserved hypothetical protein;	969.034	1198.8	834.066	1222.8
ECH_0239	riboflavin synthase, alpha subunit;	111.216	194.79	196.996	214.05
ECH_0243	hypothetical protein;	467.778	678.55	1063.62	4.4854
ECH_0246	hypothetical protein;	991.044	1416.5	1633.07	594.07
ECH_0247	conserved domain protein;	459.756	842.13	1017.8	662.32
ECH_0248	hypothetical protein;	166.083	86.602		75.746
ECH_0250	transcription-repair coupling factor;	76.5985	72.486	266.011	57.677
ECH_0251	hypothetical protein;	1042.08	1591.5	2185.27	1271.8
ECH_0252	hypothetical protein;	9827.77	13414	14493.5	9697.6
ECH_0253	hypothetical protein;	10952.4	9091.6	11464	8297.5
ECH_0254	hypothetical protein;	1395.19	1438.6	2507.66	1348.8
ECH_0255	hypothetical protein;	41.2433	57.479	241.742	39.492
ECH_0256	hypothetical protein;	76.6108	118.63	679.474	143.28
ECH_0257	hypothetical protein;	234.051	408.76	750.531	344.93

ECH_0259	hypothetical protein;	220.029	257.31		25.689
ECH_0261	hypothetical protein;	1525.25	2062.3		366.37
ECH_0263	ribonuclease HI;	1516.6	1832.6	1569.76	2074.4
ECH_0264	conserved hypothetical protein;	814.657	194.844	309.631	100.35
ECH_0266	uridylate kinase;	877.797	1041.5	536.947	976.12
ECH_0267	ribosome recycling factor;	494.75	588.65	742.052	635.83
ECH_0269	putative phosphatidate cytidylyltransferase;	436.471	423.09	498.457	495.02
ECH_0270	hypothetical protein;	39.947	42.417	258.791	44.542
ECH_0272	hypothetical protein;	77.8189	117.4	422.516	130.86
ECH_0275	conserved hypothetical protein;	15.6276	16.479		8.573
ECH_0282	hypothetical protein;	22.5963	16.496		16.735
ECH_0284	hypothetical protein;	41.9931	31.933	644.644	73.441
ECH_0289	hypothetical protein;	102.401	52.79	601.155	82.711
ECH_0290	phosphoribosylaminoimidazole-succinocarboxamide synthase;	295.007	150.19	337.245	196.72
ECH_0291	histidyl-tRNA synthetase;	433.505	343.66	578.216	282.31
ECH_0292	conserved domain protein;	2621.68	3000	3876.01	2278.3
ECH_0293	disulfide oxidoreductase;	1912.22	1879.5	838.189	1610
ECH_0294	putative cytochrome oxidase assembly protein;	120.104	91.443	250.039	118.58
ECH_0295	putative heme exporter protein CcmA;	336.403	104.99		631.46
ECH_0296	putative deoxycytidine triphosphate deaminase;	278.548	273.1	240.896	311.31
ECH_0297	conserved hypothetical protein;	278.419	209.59	244.888	255.44
ECH_0298	cold shock protein, CSD family;	8294.62	4629	3870.01	7458.6
ECH_0299	putative nitrogen regulation protein NtrY;	1432.25	442.09	314.885	720.01
ECH_0300	putative ribonuclease D;	557.588	173.78	283.469	171.05
ECH_0301	DNA ligase, NAD-dependent;	289.505	300.61	500.125	278.25
ECH_0302	glutaredoxin-related protein;	911.486	702.18	1942.87	755.2
ECH_0303	BolA family protein;	1109.59	1187	2856.02	1360.7
ECH_0304	putative thiol:disulfide oxidoreductase;	52.0668	91.297		69.639
ECH_0305	DNA repair protein RadA;	158.387	178.99	205.617	150.47
ECH_0306	TRAP transporter, 4TM/12TM fusion protein;	1386.77	1178	744.915	1198.6
ECH_0307	CvpA family protein;	517.013	316.76	395.896	446.66
ECH_0308	ribosomal protein S6;	14180.2	14663	14076.7	15027
ECH_0309	putative ribosomal protein S18;	6545.87	5164.5	3594.32	5691.3
ECH_0310	ribosomal protein L9;	5385.81	6400.4	3797.25	6018.6

ECH_0311	serine hydroxymethyltransferase;	3271.5	3754.1	2197.57	3719.8
ECH_0312	cyaY protein;	695.607	545.48	803.82	589.91
ECH_0313	ABC transporter, permease/ATP-binding protein;	346.966	340.7	590.186	268.32
ECH_0315	succinate dehydrogenase, flavoprotein subunit;	2399.91	3317.5	1504.93	3460.1
ECH_0316	succinate dehydrogenase and fumarate reductase iron-sulfur protein;	1242.58	1351.8	707.761	1487.5
ECH_0317	thymidylate synthase, flavin-dependent;	1222	1052.1	721.694	1194.5
ECH_0318	drug resistance transporter, Bcr/CflA family;	411.906	321.57	572.593	371.27
ECH_0319	holliday junction DNA helicase RuvB;	176.405	193.07	215.659	179.62
ECH_0320	holliday junction DNA helicase RuvA;	55.8992	45.341		44.845
ECH_0321	heme exporter protein CcmC;	448.921	423.8	660.742	472.71
ECH_0322	guanylate kinase;	191.262	194.41	585.36	272.3
ECH_0324	FolD bifunctional protein;	814.143	905.27	358.238	1126.8
ECH_0326	hydrolase, alpha/beta fold family;	228.885	301	578.396	308.35
ECH_0327	cytochrome C, membrane-bound;	1072.67	935.03	760.416	958.15
ECH_0328	NADH-ubiquinone/plastoquinone oxidoreductase family protein;	102.202	91.525	184.851	102.64
ECH_0329	hypothetical protein;	163.898	162.79	255.386	192.18
ECH_0330	pyruvate, phosphate dikinase;	928.704	1008.6	789.598	907.27
ECH_0331	RDD family protein;	311.56	173.51		161.92
ECH_0332	HAD-superfamily hydrolase, subfamily IA, variant 1;	1328.87	1473.2	623.469	1294.4
ECH_0334	aspartyl-tRNA synthetase;	664.555	712.78	616.69	694.52
ECH_0335	putative osmotically inducible protein;	940.446	837.98	871.135	929.58
ECH_0336	glutathione synthetase;	171.349	217.29	341.233	192.11
ECH_0337	putative cell division protein FtsQ;	237.386	113.71	134.787	141.26
ECH_0339	putative nitrogen regulation protein NtrX;	522.6	681.64	643.275	698.47
ECH_0340	glycerol-3-phosphate dehydrogenase (NAD(P)+);	114.277	169.31	303.374	114
ECH_0341	site-specific recombinase, phage integrase family;	123.03	161.59	456.202	89.065
ECH_0342	phosphoribosylformylglycinamidine cyclo-ligase;	937.533	813.36	842.516	1042.7
ECH_0343	conserved hypothetical protein;	126.502	70.36	153.312	104.15
ECH_0344	hypothetical protein;	528.225	241.01		244.21
ECH_0345	conserved hypothetical protein;	1374.45	858.68		852.03

ECH_0346	iojap-related protein;	1107.04	745.79	1582.66	1083
ECH_0347	peptidase, M48 family;	428.539	311.42	272.047	366.09
ECH_0348	conserved hypothetical protein;	368.164	397.66	977.371	278.33
ECH_0350	2-amino-4-hydroxy-6-hydroxymethyldihydropteridine-pyrophosphokinase;	514.398	285.64	570.862	434.43
ECH_0351	putative phosphoribosylformylglycinamidine synthase II;	289.767	452.89	363.711	543.88
ECH_0352	biotin synthase;	750.964	695.2	448.465	513.44
ECH_0353	hypothetical protein;	1683.27	1228.8	612.005	901
ECH_0354	conserved hypothetical protein;	607.09	695.91	439.831	604.51
ECH_0355	M23/M37 peptidase domain protein;	219.317	241.07	359.432	243.51
ECH_0356	fructose-1,6-bisphosphatase, class II;	692.47	583.44	553.151	746.44
ECH_0359	glucose inhibited division protein A;	636.739	656.13	561.54	518.19
ECH_0362	putative phosphoribosylformylglycinamidine synthase I;	415.46	485.87	506.96	539.47
ECH_0363	DNA repair protein RadC;	127.654	136.57	420.115	165.29
ECH_0364	chaperonin, 10 kDa;	6801.56	6332.3	9012.28	12150.9
ECH_0365	chaperonin, 60 kd;	6017.96	4994.1	3080.28	6977.5
ECH_0366	oxidoreductase, short-chain dehydrogenase/reductase family;	678.935	702.59	727.738	976.18
ECH_0367	ATP-dependent Clp protease, ATP-binding subunit ClpB;	849.54	1784.3	1274.05	1882.8
ECH_0368	dioxygenase family protein;	734.38	433.01	766.788	463.38
ECH_0369	cytosol aminopeptidase;	2667.92	2380.5	1502.3	2396.7
ECH_0370	phosphoribosylglycinamide formyltransferase;	800.331	809.49	443.7	798.38
ECH_0371	lipoate-protein ligase B;	107.595	142.42	210.644	156.08
ECH_0373	hypothetical protein;	477.208	569.57	519.892	580.66
ECH_0374	DNA / pantothenate metabolism flavoprotein family protein;	411.148	157.67	278.27	159.92
ECH_0376	fumarate hydratase, class II;	1640.8	1808.9	802.886	1825.7
ECH_0377	conserved domain protein;	2827.77	4400.8	2916.53	5865.3
ECH_0378	carbamoyl-phosphate synthase, large subunit;	261.525	317.05	384.462	273.57
ECH_0379	hypothetical protein;	478.807	462.18	300.207	699.23
ECH_0381	dihydropteroate synthase;	137.508	130.98	445.132	134.31
ECH_0382	conserved hypothetical protein;	146.964			59.521
ECH_0383	type I secretion system ATPase;	436.585	484.09	780.897	594.93

ECH_0385	quinone oxidoreductase;	1659.16	556.73	663.566	718.64
ECH_0387	ATP-dependent DNA helicase, UvrD/Rep family;	31.5915	31.612	137.761	41.967
ECH_0388	hypothetical protein;	773.866	1018.3	843.564	998.65
ECH_0389	ankyrin repeat protein;	672.48	296.13	627.094	346.53
ECH_0390	SPFH domain /band 7 family protein;	185.768	168.7	256.378	222.5
ECH_0392	ATPase, AFG1 family;	845.826	159.16	459.501	152.07
ECH_0395	ferrochelatase;	478.858	396.51	576.762	454.59
ECH_0396	hypothetical protein;	1773	1500.6	1873.31	1866
ECH_0397	conserved hypothetical protein;	2393.44	1433.8	1370.71	2040.1
ECH_0398	conserved hypothetical protein;	81.2633	126.76		104.14
ECH_0399	conserved hypothetical protein;	51.9351			62.269
ECH_0400	integration host factor, beta subunit;	248.431	115.98		94.833
ECH_0401	signal peptide peptidase SppA;	1207.71	1143	492.508	1076
ECH_0402	putative ribosomal protein S1;	2522.84	2899.6	1807.28	2834.7
ECH_0403	cytidylate kinase;	105.722	121.4	171.318	105.88
ECH_0404	RNA methyltransferase, TrmH family, group 3;	156.745	150.37	161.957	139.25
ECH_0408	ribosomal protein S10;	1455.28	2048.7	910.121	2106.2
ECH_0409	ribosomal protein L3;	2484.01	3059.6	1515.19	3167.1
ECH_0410	ribosomal protein L4;	1727.63	1957.8	1549.39	2006.1
ECH_0411	ribosomal protein L23;	2660.02	1584.7	2178.82	1889.3
ECH_0412	ribosomal protein L2;	2554.93	3853.3	1432.54	3783.1
ECH_0413	ribosomal protein S19;	1811.91	1566.2	734.158	1736.9
ECH_0414	ribosomal protein L22;	1225.24	1749.9	1146.3	1907.3
ECH_0415	ribosomal protein S3;	972.467	1473.5	1159.68	1573.4
ECH_0416	ribosomal protein L16;	855.402	1282.3	739.852	1485.1
ECH_0418	ribosomal protein S17;	518.451	1013.3	737.781	1260.3
ECH_0419	ribosomal protein L14;	643.995	1159.8	1725.27	1322.8
ECH_0420	ribosomal protein L24;	395.179	612.96	470.529	775.07
ECH_0421	ribosomal protein L5;	2559.22	2828.8	1768.89	3208.6
ECH_0422	ribosomal protein S14p;	1874.16	1792.1	634.291	2173.4
ECH_0423	ribosomal protein S8;	1955.3	2127.8	1070.19	2299.3
ECH_0424	ribosomal protein L6;	1840.53	1802.2	746.975	2175.2
ECH_0425	ribosomal protein L18;	1860.72	2056	1237.39	2584.5
ECH_0426	ribosomal protein S5;	2295.14	2327.6	1786.89	2663.9

ECH_0427	ribosomal protein L15;	1585.16	1314	728.021	1525.2
ECH_0428	preprotein translocase, SecY subunit;	979.56	805.02	425.038	845.42
ECH_0429	adenylate kinase;	870.263	1003.1	874.293	913.7
ECH_0430	ribosomal protein S13;	7384.34	5577.2	4974.07	5379.8
ECH_0431	ribosomal protein S11;	4707.86	6278.4	3820.73	5717.4
ECH_0432	DNA-directed RNA polymerase, alpha subunit;	7468.67	4811.2	5624.79	5015.3
ECH_0433	ribosomal protein L17;	3003.07	2418.9	2961.29	2821.1
ECH_0434	phenylalanyl-tRNA synthetase, beta subunit;	913.41	986.68	983.995	977.33
ECH_0435	ComEC/Rec2-related protein;	45.9458	56.36	223.646	49.845
ECH_0438	sodium:alanine symporter family protein;	1049.56	755.81	798.186	892.61
ECH_0439	hypothetical protein;	643.995	768.08	880.608	1314.2
ECH_0441	4-hydroxybenzoate octaprenyltransferase;	279.455	218.2	258.495	248.92
ECH_0442	putative flavin reductase;	2582.14	2504.3	1006.41	3232.1
ECH_0443	dihydrodipicolinate reductase;	3714.48	3728.8	2076.12	5239.2
ECH_0444	phosphate ABC transporter, ATP-binding protein;	3422.81	2906.6	1927.35	3382.1
ECH_0445	queuine tRNA-ribosyltransferase;	83.8824	123.87	239.018	118.08
ECH_0446	ribosomal protein L32;	1869.66	2748.7	1182.65	2698.8
ECH_0447	fatty acid/phospholipid synthesis protein PlsX;	1810.29	1849.3	906.023	1870.9
ECH_0448	3-oxoacyl-(acyl-carrier-protein) synthase III;	397.995	606.99	411.1	594.17
ECH_0449	cytidine and deoxycytidylate deaminase family protein;	243.391	173.29		154.13
ECH_0450	conserved hypothetical protein;	1261.59	904.08	3709.94	1293
ECH_0451	replicative DNA helicase;	313.472	314.35	502.763	384.77
ECH_0452	FAD-dependent oxidoreductase;	124.598	129.82	473.842	162.57
ECH_0454	conserved hypothetical protein;	98.1597	55.121		70.563
ECH_0455	putative geranyltranstransferase;	367.085	325.45	249.456	299.31
ECH_0459	ribonucleoside-diphosphate reductase, alpha subunit;	260.606	383.5	535.529	376.62
ECH_0460	conserved hypothetical protein TIGR00250;	64.7665	106.38	686.812	78.68
ECH_0461	adenylosuccinate synthetase;	1403.16	1276.6	820.605	1415.5
ECH_0462	OmpA family protein;	3901.23	3708	2352.64	3850.9
ECH_0464	putative carboxypeptidase;	656.792	664.66	646.977	602.7
ECH_0465	transketolase;	373.273	516.11	470.942	456.1
ECH_0466	monovalent cation/proton antiporter, MrpF/PhaF subunit family;	432.501	252.24	711.884	386.51

ECH_0467	monovalent cation/proton antiporter, MnhG/PhaG subunit family;	1095.64	751.58	603.845	1074.1
ECH_0469	Na(+)/H(+) antiporter subunit C;	137.025	52.469		95.547
ECH_0470	ribonuclease, Rne/Rng family;	1220.54	1056.3	598.126	1054.6
ECH_0471	chaperone protein DnaK;	2933.79	2944.6	1858.19	3820.9
ECH_0472	hypothetical protein;	768.274	982.86	623.015	1275.6
ECH_0473	aromatic-rich protein family;	793.453	306.14	445.231	548.06
ECH_0474	NADH-ubiquinone/plastoquinone oxidoreductase family protein;	127.584	75.864	264.487	94.101
ECH_0475	signal recognition particle protein;	979.813	899.63	444.765	1013.8
ECH_0476	GTP-binding protein Era;	756.634	700.85	479.242	675.77
ECH_0477	conserved hypothetical protein;	520.39	513.25	414.128	601.62
ECH_0478	conserved hypothetical protein;	236.575	207.39	398.907	243.23
ECH_0479	metallopeptidase, M24 family;	276.756	273.25	577.591	276.14
ECH_0480	uroporphyrinogen-III synthase;	319.789	92.24	103.141	53.612
ECH_0482	putative lipoprotein;	60.2278	67.568	418.047	70.529
ECH_0483	primosomal protein N';	158.096	146.43	77.885	130.54
ECH_0484	50S ribosomal protein L28;	7585.98	4697.6	4206.42	4963.9
ECH_0485	diaminopimelate decarboxylase;	537.509	172.79	194.657	223.96
ECH_0486	ribosomal large subunit pseudouridine synthase, RluA family;	64.6802	64.97	108.468	51.921
ECH_0487	propionyl-CoA carboxylase, alpha subunit;	872.679	823.68	611.472	714.35
ECH_0488	hypothetical protein;	314.965	311.33	683.026	292.13
ECH_0489	hypothetical protein;	504.506	527.5	573.511	447.79
ECH_0490	lipoic acid synthetase;	2252.9	1956.4	1027.64	1846.9
ECH_0492	putative phosphate ABC transporter, permease protein;	118.991	116.26	188.638	110.46
ECH_0493	superoxide dismutase, Fe;	1147.64	1052.5	565.32	1375.4
ECH_0494	type IV secretion system protein VirB3;	2326.51	785.85	1034.28	823.25
ECH_0495	type IV secretion system protein VirB4;	942.62	1492	516.935	1034.9
ECH_0496	type IV secretion system protein VirB6;	1059.05	737.89	435.49	504.87
ECH_0497	type IV secretion system protein, VirB6 family;	303.783	259.7	235.987	229.5
ECH_0498	type IV secretion system protein,VirB6 family;	1154.89	553.31	490.334	483.78
ECH_0499	type IV secretion system protein,VirB6 family;	1129.9	276.45	558.103	224.67
ECH_0500	conserved hypothetical protein;	39.4023	30.929		25.102
ECH_0501	deoxyuridine 5'triphosphate nucleotidohydrolase;	754.478	576.95	525.323	721.97

ECH_0502	hydroxymethylbutenyl pyrophosphate reductase;	437.715	400.15	350.446	404.88
ECH_0503	carbamoyl-phosphate synthase, small subunit;	440.765	444.82	446.391	364.62
ECH_0504	GTP-binding protein EngA;	859.313	288.6	623.123	222.43
ECH_0505	2,3-bisphosphoglycerate-independent phosphoglycerate mutase;	80.0098	103.51	158.952	106.98
ECH_0508	PQQ enzyme repeat family protein;	1715.41	1620.4	1081.1	1880.8
ECH_0509	dihydrolipoamide dehydrogenase;	1153.25	1257.2	673.934	1673.2
ECH_0510	efflux transporter, RND family, MFP subunit;	807.236	935.63	762.916	1116
ECH_0511	translation initiation factor IF-1;	1402.74	1404.9	987.354	1828.1
ECH_0512	maf protein;	555.834	812.49	1083.88	1077.6
ECH_0514	ribosomal protein S2;	1215.63	1138.7	776.074	1639.9
ECH_0515	translation elongation factor Ts;	1968.56	941.79	910.394	1304.4
ECH_0516	conserved hypothetical protein;	170.173	33.23		45.13
ECH_0517	putative cation ABC transporter, permease protein;	503.942	52.239		47.571
ECH_0518	putative membrane protein;	14.7998			16.741
ECH_0519	hypothetical protein;	768.403			109.09
ECH_0520	ubiquinol-cytochrome c reductase, iron-sulfur subunit;	4210.67	4246.2	2821.92	4237.4
ECH_0521	ubiquinol-cytochrome c reductase, cytochrome b;	1989.54	1874.7	1044.02	1725.6
ECH_0522	ubiquinol-cytochrome c reductase, cytochrome c1;	1130.37	1002.4	886.503	1109
ECH_0523	conserved domain protein;	1525.68	159.08	501.737	141.46
ECH_0525	conserved domain protein;	1055.64	1240.9	426.791	1997.4
ECH_0526	conserved domain protein;	424.364	336.11	552.191	472.26
ECH_0528	thiamine-monophosphate kinase;	38.6139	39.151		40.206
ECH_0529	YihY family protein;	121.096	88.851	321.528	92.24
ECH_0531	hypothetical protein;	1363.53	518.42	11788.09	402.6
ECH_0532	magnesium chelatase, subunit D/I family, ComM subfamily;	28.4145	20.373		26.854
ECH_0533	ribosomal RNA large subunit methyltransferase J;	342.525	246.85	323.995	279.84
ECH_0535	hypothetical protein;	864.581	788.94	1603.03	775.73
ECH_0536	putative DNA repair protein RecO;	59.1424	34.085		40.713
ECH_0537	arginyl-tRNA synthetase;	508.417	527.99	642.866	500.33
ECH_0538	isoleucyl-tRNA synthetase;	729.498	389.44	355.271	526.34

ECH_0539	antioxidant, AhpC/TSA family;	2772.25	2071	1136.58	3032.4
ECH_0540	conserved hypothetical protein;	1323.56	1490.2	659.453	1843.2
ECH_0541	5-formyltetrahydrofolate cyclo-ligase family protein;	251.457	124.43	210.701	185.01
ECH_0542	S-adenosyl-methyltransferase MraW;	332.689	283.34	578.95	310.34
ECH_0543	GTP-binding protein, GTP1/Obg family;	202.77	191.38	252.972	210.05
ECH_0544	enolase;	490.345	583.4	597.917	654.42
ECH_0545	ribosomal protein L21;	1199.47	1402.3	816.573	1624.1
ECH_0546	ribosomal protein L27;	1093.95	1605.5	1211.57	2019.1
ECH_0548	NADH dehydrogenase I, F subunit;	617.703	768.17	740.698	880.36
ECH_0549	hypothetical protein;	67.4431	89.947	1089.3	93.244
ECH_0551	hypothetical protein;	66.2001	96.654		44.268
ECH_0552	NADH dehydrogenase I, J subunit;	1393.72	667.82	1137.31	656.08
ECH_0553	NADH dehydrogenase I, K subunit;	489.759	195.79	415.49	221.2
ECH_0554	NADH dehydrogenase I, L subunit;	291.4	259.57	488.873	226.46
ECH_0555	NADH dehydrogenase I, M subunit;	443.289	293.96	409.93	265.5
ECH_0556	NADH dehydrogenase I, N subunit;	169.292	93.446	156.009	106.16
ECH_0557	1-deoxy-D-xylulose 5-phosphate reductoisomerase;	331.062	366.04	484.815	340.36
ECH_0558	putative lipoprotein;	414.395	431.63	550.226	476.5
ECH_0559	4-hydroxy-3-methylbut-2-en-1-yl diphosphate synthase;	624.981	757.39	489.178	663.64
ECH_0560	Sec-independent protein translocase protein TatC;	326.404	209.08	396.813	225.92
ECH_0561	AcrB/AcrD/AcrF family protein;	248.794	187.13	331.944	159.23
ECH_0562	N utilization substance protein A;	2359.03	2776.5	3447.21	2328.6
ECH_0563	translation initiation factor IF-2;	837.572	1090.9	1118.52	875.57
ECH_0564	ribosome-binding factor A;	271.892	77.995		123.88
ECH_0567	ATP-dependent Clp protease, ATP-binding subunit ClpA;	626.561	566.09	177.386	534.58
ECH_0568	phage minor structural protein, N-terminal domain protein;	65.6748	81.803	807.067	79.641
ECH_0569	hypothetical protein;	101.683			42.037
ECH_0570	hypothetical protein;	13247.1		15176	10916
ECH_0571	magnesium transporter;	203.589	183.41	311.455	147.73
ECH_0573	ATP synthase F1, beta subunit;	4397.11	4765.4	1536.78	5403
ECH_0574	ATP synthase F1, epsilon subunit;	1929.23	817.59	1203.68	1253.2

ECH_0575	putative transaldolase;	1205.48	1112.6	839.216	1489.6
ECH_0576	conserved hypothetical protein;	968.155	875.23	1353.6	1216.9
ECH_0577	RmuC family protein;	75.3683	48.181		73.803
ECH_0579	type IV secretion system protein VirB8;	171.314	25.55		41.741
ECH_0581	sodium:dicarboxylate symporter family protein;	179.086	106.91	102.424	122.99
ECH_0584	seryl-tRNA synthetase;	120.258	142.69	227.275	125.88
ECH_0585	conserved domain protein;	475.625	249.72	228.971	252.11
ECH_0587	conserved hypothetical protein;	1988.81	1526.2	831.626	1621.6
ECH_0588	tRNA delta(2)-isopentenylpyrophosphate transferase;	158.044	129.21	184.445	139
ECH_0591	coproporphyrinogen III oxidase, aerobic;	134.686	167.13	272.411	168.43
ECH_0593	conserved hypothetical protein;	1082.54	914.15	1295.08	1176.6
ECH_0594	acetylglutamate kinase;	300.787	346.75	254.504	390.46
ECH_0595	GTP-binding protein EngB;	110.031	55.396		57.184
ECH_0597	peptide chain release factor 1;	320.585	304.14	515.185	268.33
ECH_0599	propionyl-CoA carboxylase, beta subunit;	664.623	844.95	700.575	983.67
ECH_0600	hypothetical protein;	74.1441	341.51		366.99
ECH_0601	hypothetical protein;	13.5578		57.5091	7.1636
ECH_0602	formamidopyrimidine-DNA glycosylase;	84.7952	23.967	111.411	60.451
ECH_0603	hypothetical protein;	136.405			163.26
ECH_0605	glutamyl-tRNA synthetase;	114.123	102.66	215.659	144.39
ECH_0607	hypothetical protein;	42.4992	33.515	106.828	43.016
ECH_0609	hypothetical protein;	2533.67	2960.8	6776.83	2786.2
ECH_0611	hypothetical protein;	27.6314	52.445		44.134
ECH_0612	hypothetical protein;	799.115	955.99	711.984	850.53
ECH_0615	NADH dehydrogenase I, E subunit;	405.057	450.34	533.223	456.96
ECH_0616	NADH dehydrogenase I, D subunit;	1050.38	1099.8	519.99	911.62
ECH_0617	NADH dehydrogenase I, H subunit;	974.696	829.54	785.28	1015.6
ECH_0618	NADH dehydrogenase I, G subunit;	1393.31	1428.9	986.861	1382.4
ECH_0619	conserved hypothetical protein;	870.577	1305	1358.95	1641.8
ECH_0620	DNA gyrase, B subunit;	956.065	1008.4	585.707	1305.3
ECH_0621	aspartate carbamoyltransferase;	112.699	97.942	165.339	112
ECH_0622	tRNA pseudouridine synthase A;	33.0678	31.432		32.939
ECH_0625	putative lipoprotein;	1142.04	1059.4	965.637	1318.2
ECH_0626	lysyl-tRNA synthetase;	1350.98	1114.5	1453.17	1266.7

ECH_0627	conserved hypothetical protein;	3437.62	2605.2	2586.12	2698.3
ECH_0628	rrf2/aminotransferase, class V family protein;	1298.89	1084.1	1207.34	895.51
ECH_0629	cysteine desulfurase;	1593.91	1441.1	1390.5	1219.5
ECH_0630	FeS cluster assembly scaffold IscU;	732.67	1340.3	1687.77	1333.4
ECH_0631	iron-sulfur cluster assembly accessory protein;	745.449	572.61	568.291	605.82
ECH_0632	co-chaperone Hsc20;	340.09	152.56	320.574	97.301
ECH_0633	chaperone protein HscA;	582.869	497.97	600.216	382.7
ECH_0634	iron-sulfur cluster binding protein;	434.544	438.82	239.621	373.01
ECH_0635	hypothetical protein;	171.839	89.85	216.864	65.996
ECH_0636	cytochrome c biogenesis family protein;	52.7247	27.493		33.11
ECH_0637	3-demethylubiquinone-9 3-methyltransferase;	1423.36	1059.5	1265.44	1285
ECH_0638	ribose 5-phosphate isomerase B;	3274	3259	2156.59	3883.1
ECH_0639	hypothetical protein;	1035.9	568.32	1698.31	991.69
ECH_0640	conserved domain protein;	396.771	497.38	590.148	545.12
ECH_0641	malate dehydrogenase, NAD-dependent;	2556.66	3796.2	2513.73	4193
ECH_0642	dihydroneopterin aldolase;	84.7361	63.445		57.024
ECH_0643	RDD family protein;	525.278	230.39	308.084	292.2
ECH_0644	putative metalloendopeptidase, glycoprotease family;	1092.15	438.31	764.049	492.79
ECH_0645	immunogenic protein;	1031.65	1148	833.529	968.1
ECH_0646	triosephosphate isomerase;	1415.55	1581.1	885.902	1356
ECH_0648	dimethyladenosine transferase;	399.456	138.68	341.331	113.42
ECH_0649	pyridine nucleotide-disulphide oxidoreductase family protein;	170.222	185.21	178.125	200.84
ECH_0650	pmbA protein;	601.834	586.9	485.113	511.25
ECH_0651	GTP cyclohydrolase I;	1527.91	1399.1	779.082	1181.3
ECH_0652	ATP synthase F1, gamma subunit;	2852.18	2021.4	2133.65	1970.2
ECH_0653	ankyrin repeat protein;	410.187	533.34	790.85	485.7
ECH_0654	putative ribosomal subunit interface protein;	299.144	178.08	261.405	179.27
ECH_0655	RNA polymerase sigma-32 factor;	1840.66	2155.7	2763.33	1987.6
ECH_0656	ribosomal protein L36;	685.772	1388.2	1203.68	1499.9
ECH_0660	conserved hypothetical protein;	267.844	129.309	23.314	9.069
ECH_0663	conserved hypothetical protein;	25.0452			24.08
ECH_0664	hypothetical protein;	80.478	40.41		58.312
ECH_0665	phage uncharacterized protein;	34.0747	31.587	307.429	30.593
ECH_0666	adenosylmethionine-8-amino-7-oxononanoate	328.625	324.5	378.792	291.61

	aminotransferase;				
ECH_0667	proline dehydrogenase/delta-1-pyrroline-5-carboxylate dehydrogenase;	701.08	1135.9	831.68	1276.2
ECH_0669	3-oxoacyl-(acyl-carrier-protein) reductase;	1230.04	979.01	991.336	1274.3
ECH_0670	putative membrane protein;	376.076	319.72	557.321	411.77
ECH_0673	putative membrane protein;	143.425	63.784	251.742	129.72
ECH_0675	exodeoxyribonuclease III;	699.524	664.65	497.086	1123.9
ECH_0676	arginine biosynthesis bifunctional protein ArgJ;	5470.9	4988.3	2662.52	6311
ECH_0678	hypothetical protein;	37.416	20.084		54.142
ECH_0679	hypothetical protein;	73.8725	39.653		44.227
ECH_0680	argininosuccinate synthase;	734.237	801.49	667.777	948.72
ECH_0681	hypothetical protein;	4787.59	2668	3060.03	4189.6
ECH_0682	hypothetical protein;	1861.81	775.41	1883.22	1482.7
ECH_0684	ankyrin repeat protein;	1765.23	3651.1	1309.57	2321.3
ECH_0685	hypothetical protein;	1585.96	1311	2027.84	1008.6
ECH_0688	putative deoxyguanosinetriphosphate triphosphohydrolase;	452.849	453.84	465.997	440.44
ECH_0689	iron-sulfur cluster assembly accessory protein;	17663.3	11067	8783.46	12757
ECH_0690	signal peptidase I;	5614.13	5345.8	5656.48	5498.5
ECH_0691	NADH dehydrogenase I, I subunit;	2447.88	2383.4	2892.37	2089.5
ECH_0692	hypothetical protein;	68.3356			48.3
ECH_0693	GTP-binding protein TypA;	2009.28	2006.6	1788.39	2316.2
ECH_0695	hypothetical protein;	761.758	927.88	934.768	8.8077
ECH_0696	hypothetical protein;	390.18	453.15		530.86
ECH_0697	hypothetical protein;	8509.63	8434.7	7287.48	358.45
ECH_0698	hypothetical protein;	319.974	260.05	321.879	6817.1
ECH_0699	hypothetical protein;	719.648	714.26	1225.69	326.89
ECH_0700	hypothetical protein;	2670.86	1714.7	1072.71	1362.8
ECH_0701	porphobilinogen deaminase;	326.475	496.52	577.014	388.32
ECH_0702	folylpolyglutamate synthase;	4118.64	4220.8	4570.39	3853.7
ECH_0703	glutamyl-tRNA(Gln) amidotransferase, A subunit;	641.251	662.95	521.471	545.38
ECH_0704	conserved hypothetical protein;	191.932	166.45	320.457	173.85
ECH_0705	peptide chain release factor 2, programmed frameshift;	3364.17	3882.4	2913.29	4150.5
ECH_0707	hypothetical protein;	6790.2	6074.5	8831.37	4631.6

ECH_0714	hypothetical protein;	767.085	336.42		311.75
ECH_0715	hypothetical protein;	1449.26	1567.2	1449.45	881.52
ECH_0716	hypothetical protein;	35.23	33.619		28.242
ECH_0720	hypothetical protein;	112.052	113.95	366.053	118.5
ECH_0721	putative lipoprotein;	295.882	316.05	718.863	392.25
ECH_0722	hypothetical protein;	29.2806	45.341		38.966
ECH_0724	GTP-binding protein LepA;	599.66	778.65	878.477	695.94
ECH_0725	conserved hypothetical protein;	648.078	280.11	520.837	484.67
ECH_0726	polyribonucleotide nucleotidyltransferase;	3812.81	4390.6	2605.77	4787.4
ECH_0727	ribosomal protein S15;	3986.2	5021.1	3946.1	6005.1
ECH_0728	tRNA pseudouridine synthase B;	86.448	136.42	697.08	99.716
ECH_0730	glycoprotease family protein;	168.608	157.8	594.163	171.21
ECH_0731	rotamase family protein;	879.921	641.48	485.657	712.87
ECH_0732	aspartate aminotransferase;	962.679	834.86	475.639	1048
ECH_0734	antioxidant, AhpC/Tsa family;	4875.62	3822.6	3130.2	4262.3
ECH_0735	thioredoxin-disulfide reductase;	1143.54	1214.9	1270.97	1330.3
ECH_0737	pantetheine-phosphate adenylyltransferase;	768.751	415.49	1416.07	463.07
ECH_0739	conserved hypothetical protein;	929.304	959.6	1335.03	782.69
ECH_0740	prolyl-tRNA synthetase;	2035.46	1857.2	1273.66	1879.6
ECH_0741	holo-(acyl-carrier-protein) synthase;	53.3018			88.787
ECH_0744	conserved hypothetical protein;	3042.46	2051.5	9008.54	2234.7
ECH_0745	conserved domain protein;	546.869	436.65	2573.41	397.27
ECH_0750	DNA topoisomerase I;	340.286	362.41	514.85	291.77
ECH_0751	YjeF family protein;	752.216	846.55	1203.79	949.91
ECH_0752	hypothetical protein;	503.333	340.22	992.031	346.58
ECH_0753	conserved hypothetical protein;	1932.5	1587.5	4153.22	1714.8
ECH_0754	O-methyltransferase;	105.15	77.323		70.561
ECH_0755	sensor histidine kinase/response regulator;	111.005	77.23	223.723	67.014
ECH_0756	divalent ion tolerance protein CutA1;	815.655	153.14		154.13
ECH_0757	4-diphosphocytidyl-2C-methyl-D-erythritol kinase;	707.831	666.67	960.178	910.8
ECH_0758	putative tRNA-dihydrouridine synthase;	74.344	126.44	732.427	101.65
ECH_0760	RNA polymerase sigma factor RpoD;	2041.42	1939.7	1329.26	1807.1
ECH_0761	DNA primase;	134.657	119.2	347.837	117.68
ECH_0762	conserved hypothetical protein;	121.808	138.08	365.85	128.05

ECH_0763	conserved domain protein;	1802.43	1302.4	2715.22	1330.2
ECH_0764	conserved hypothetical protein;	966.551	907.48	1421.93	798.26
ECH_0765	hypothetical protein;	190.656			60.002
ECH_0766	ribonucleoside-diphosphate reductase, beta subunit;	1189.7	947.86	1008.62	1098.2
ECH_0767	conserved hypothetical protein;	92.7738	97.959	284.309	84.855
ECH_0768	cysteinyl-tRNA synthetase;	634.416	642.79	595.406	701.12
ECH_0769	exopolysaccharide synthesis protein;	1278.31	905.78	564.821	942.11
ECH_0771	NAD-glutamate dehydrogenase family protein;	250.678	257.16	341.732	248.12
ECH_0772	hypothetical protein;	113.582	131.61		79.644
ECH_0773	putative response regulator/diguanylate cyclase;	164.488	241.91	202.033	202.25
ECH_0774	ribosomal large subunit pseudouridine synthase C;	112.248	89.373	350.096	118.25
ECH_0776	putative inositol monophosphatase;	287.411	261.32	510.54	259.38
ECH_0777	translation elongation factor P;	1768.76	2620.8	2054.44	2707.3
ECH_0778	conserved hypothetical protein;	67.5346	70.976	293.129	59.022
ECH_0779	phosphatidylserine decarboxylase;	3257.68	3466.3	2071.09	3299.8
ECH_0780	putative CDP-diacylglycerol--serine O-phosphatidyltransferase;	700.791	497.97	349.717	602.51
ECH_0781	sodium:alanine symporter family protein;	156.054	157.67	271.328	133.69
ECH_0782	inner-membrane protein, 60 kDa;	522.004	390.88	494.528	338.53
ECH_0783	6,7-dimethyl-8-ribityllumazine synthase;	1315.1	1441.3	833.881	1555.9
ECH_0784	putative N utilization substance protein B;	772.794	406.5	271.114	455.36
ECH_0785	excinuclease ABC, A subunit;	265.307	288.07	302.64	228.71
ECH_0786	NADH dehydrogenase I, A subunit;	2412.93	1164.8	1739.19	1299.7
ECH_0787	NADH dehydrogenase I, B subunit;	1004.12	979.42	814.712	1175.5
ECH_0788	NADH dehydrogenase I, C subunit;	524.223	238.78	368.743	317.4
ECH_0789	cytochrome c-type biogenesis protein CcmE;	1154.93	363.81	531.101	290.52
ECH_0790	orotidine 5'-phosphate decarboxylase	293.752			230.47
ECH_0791	acid phosphatase SurE;	723.06	1020.5	839.14	1167.2
ECH_0792	orotidine 5'-phosphate decarboxylase;	346.249	493.27	734.356	596.42
ECH_0793	hydrolase, TatD family;	474.328	527.34	743.652	468.98
ECH_0794	leucyl-tRNA synthetase;	1593.43	306.81	582.019	393.16
ECH_0795	conserved hypothetical protein;	382.479	629.93	534.201	567.45
ECH_0796	DNA-directed RNA polymerase, omega subunit;	1671.56	1473	1380.84	1859.6
ECH_0797	Smr domain protein;	94.9937	107.44		85.991

ECH_0798	thiamin biosynthesis protein ThiC;	1111.65	1134.6	796.578	1351
ECH_0801	dephospho-CoA kinase;	115.208	118.84		94.91
ECH_0802	Sua5/YciO/YrdC/YwlC family protein;	48.2344	77.72	199.07	83.334
ECH_0803	conserved hypothetical protein;	41.223	50.155		57.28
ECH_0804	DNA-binding protein HU;	3113.23	3425	1292.5	4204.7
ECH_0805	pyridoxal phosphate biosynthesis protein PdxJ;	2245.51	1909.9	1473.67	2163.6
ECH_0806	nicotinate (nicotinamide) nucleotide adenylyltransferase;	224.461	235.26	676.802	231.9
ECH_0807	hypothetical protein;	600.994	620.43	759.635	746.94
ECH_0809	chromosomal replication initiator protein DnaA;	1386.94	1320.3	904.376	1511.7
ECH_0810	hypothetical protein;	403.013	324.38		326.53
ECH_0811	enoyl-(acyl-carrier-protein) reductase;	3875.96	4025.4	2164.67	4335.4
ECH_0813	glutamyl-tRNA(Gln) amidotransferase, B subunit;	1419.24	1520.8	917.222	1715.3
ECH_0814	putative malonyl-CoA decarboxylase;	609.989	445.03	395.532	607.69
ECH_0815	single-strand binding protein;	473.021	356.27	409.479	467.42
ECH_0816	major facilitator family transporter;	309.874	252.85	550.954	255.66
ECH_0818	major facilitator family transporter;	373.944	274.18	1222.38	229.98
ECH_0820	conserved hypothetical protein;	409.514	65.915	167.342	73.542
ECH_0821	delta-aminolevulinic acid dehydratase;	305.51	373.57	220.856	355.89
ECH_0822	glutamine-dependent NAD(+) synthetase;	62.2961	45.232		38.903
ECH_0824	DNA mismatch repair protein MutS;	309.471	314.29	573.305	312.34
ECH_0825	hypothetical protein;	3431.48	3640.1	3435.83	1904.1
ECH_0826	HIT family protein;	1512.21	1477.7	1622.05	921.42
ECH_0828	dihydrodipicolinate synthase;	2806.53	2652.9	940.794	2829
ECH_0829	hypothetical protein;	104.999			272.85
ECH_0830	phage major capsid protein, HK97 family;	397.034	123.75	202.18	242.55
ECH_0832	2-oxoglutarate dehydrogenase, E1 component;	1303.06	1436.4	1188.13	1122.8
ECH_0833	hypothetical protein;	907.175	151.61		235.73
ECH_0834	conserved hypothetical protein;	747.373	525.8	1250.82	703.29
ECH_0835	conserved hypothetical protein;	33.6452	28.428		27.848
ECH_0836	hypothetical protein;	31.9347	26.761	208.123	28.156
ECH_0837	tRNA-i(6)A37 modification enzyme MiaB;	565.8	566.22	662.068	455.64
ECH_0838	conserved hypothetical protein;	24.419	26.663	254.966	26.358
ECH_0840	2-polyprenylphenol 6-hydroxylase;	935.55	235.34	295.915	257.82
ECH_0841	putative cation ABC transporter, ATP-binding	288.731	123.46	319.495	155.31

	protein;				
ECH_0842	putative cation ABC transporter, periplasmic cation-binding protein;	324.273	242.62		206.11
ECH_0843	recombination protein RecR;	894.917	1095.1	693.19	1313.1
ECH_0844	twin-arginine translocation protein, TatA;	735.866	1215.5	718.863	1632.9
ECH_0845	ABC transporter, ATP-binding protein;	579.983	633.1	714.647	629.32
ECH_0846	conserved hypothetical protein;	168.487	165.44	150.46	192.54
ECH_0847	glutathione S-transferase family protein;	577.122	401.24	262.288	412.41
ECH_0848	biotin--acetyl-CoA-carboxylase ligase;	1015.07	253.6	299.526	231.38
ECH_0849	hypothetical protein;	1439.25	1435.2	1064.19	899.68
ECH_0852	adenylosuccinate lyase;	177.3	148.57	348.64	157.44
ECH_0853	chaperone protein HtpG;	1637.61	1538.2	1152.66	1871.5
ECH_0854	conserved hypothetical protein;	816.143	978.34	942.089	1130.3
ECH_0856	endonuclease III;	88.4203	193.49		58.877
ECH_0857	endonuclease III;	638.628	311.1	245.299	252.96
ECH_0858	DNA gyrase, A subunit;	3263.43		2007.86	3483.8
ECH_0860	helicase, UvrD/Rep family;	157.689	185.58	370.664	156.71
ECH_0862	hypothetical protein;	4653.15	4549.8	3635.24	3626.2
ECH_0864	conserved domain protein;	455.888	246.1	273.646	252.46
ECH_0865	hypothetical protein;	24.3302	24.498	476.87	24.49
ECH_0866	hypothetical protein;	25.344	47.655	299.707	36.122
ECH_0871	putative twitching motility protein PilT;	549.03	278.18	740.131	344.29
ECH_0872	tRNA (5-methylaminomethyl-2-thiouridylate)-methyltransferase;	264.57	271.47	480.545	246.58
ECH_0873	Coq7 family protein;	655.59	567.94	403.572	625.55
ECH_0874	disulfide bond formation family protein;	321.783	185.97	382.181	186.05
ECH_0875	putative phosphatidylglycerophosphatase A;	434.506	258.88	266.011	266.66
ECH_0876	ribosomal protein L33;	8272.92	2799.9	3556.48	3384.4
ECH_0877	ankyrin repeat protein;	1372.73	1538.7	1427.05	1697.1
ECH_0878	hypothetical protein;	217.54	55.823	1125.63	45.327
ECH_0879	hypothetical protein;	520.531	153.565	240.502	129.2
ECH_0880	hypothetical protein;	378.914			100.96
ECH_0881	acyl carrier protein;	1066.57	907.7	867.324	1171.6
ECH_0882	3-oxoacyl-(acyl-carrier-protein) synthase II;	694.716	608.35	841.223	735.95
ECH_0884	DNA mismatch repair protein, MutL/HexB family;	362.832	385.93	431.958	343.09

ECH_0885	sensor histidine kinase;	164.615	153	279.304	132.72
ECH_0886	acetylornithine/succinyldiaminopimelate aminotransferase;	3243.91	3256.6	2244.39	3224.4
ECH_0888	putative membrane protein;	2867.5	3142.3	7373.05	2554.5
ECH_0890	putative cell division protein FtsK;	429.207	491.21	856.906	490.46
ECH_0891	YGGT family protein;	66.897	35.813		84.368
ECH_0892	CBS domain protein;	138.819	125.43	124.228	117.81
ECH_0893	thiamine-phosphate pyrophosphorylase;	73.6686	73.22	724.111	64.08
ECH_0895	conserved hypothetical protein;	847.052	669.38		727.92
ECH_0896	rhodanese domain protein;	2973.13	2324.2	1859.67	2606.4
ECH_0897	methionyl-tRNA formyltransferase;	356.226	254.35	312.138	305.73
ECH_0899	ATP-dependent protease La;	1450.54	1481.8	856.728	1440
ECH_0900	ATP-dependent Clp protease, ATP-binding subunit ClpX;	1748.85	1962.9	921.982	2005.2
ECH_0901	ATP-dependent Clp protease, proteolytic subunit ClpP;	2516.66	2817.7	1024.38	3059.6
ECH_0902	trigger factor;	1306.86	1174.6	1225.34	1104.5
ECH_0905	phosphatidylglycerophosphatase A;	567.08	717.41	957.067	726.37
ECH_0906	acetyltransferase, GNAT family;	425.747	338.21	447.098	277.21
ECH_0907	conserved hypothetical protein;	433.219	277.72	485.515	307.29
ECH_0908	conserved hypothetical protein;	956.127	2160.4	1480.64	2473
ECH_0909	hypothetical protein;	59.1992		329.164	19.304
ECH_0910	conserved hypothetical protein;	397.368	238.48	3070.22	298.71
ECH_0912	ribosomal protein S4;	5230.68	4561.7		4996.6
ECH_0913	conserved hypothetical protein;	570.646	114.369		51.68
ECH_0914	phosphomethylpyrimidine kinase;	103.566	135.17	231.634	119.56
ECH_0916	hypothetical protein;	483.66	667.07	1173.06	483.43
ECH_0917	putative succinate dehydrogenase, hydrophobic membrane anchor protein;	23.4983	33.788		37.932
ECH_0918	succinate dehydrogenase, cytochrome b556 subunit;	490.681	249.35	334.355	285.4
ECH_0920	putative 3-phosphoshikimate 1-carboxyvinyltransferase;	65.7742	57.677	246.324	76.528
ECH_0921	hypothetical protein;	81.5385			85.999
ECH_0922	DNA polymerase III, alpha subunit;	413.4	493.05	459.79	503.81
ECH_0925	hypothetical protein;	67.7889			56.761
ECH_0928	hypothetical protein;	140.484			59.684

ECH_0929	putative lipoprotein;	2898.72	3375	3191.44	3751.8
ECH_0930	putative bolA protein;	1643.18	251.9	1245.04	309.18
ECH_0931	pyridoxamine 5'-phosphate oxidase;	191.927	287.64	560.713	284.16
ECH_0935	phospholipase/carboxylesterase family protein;	381.313	354.1	372.868	452.22
ECH_0936	hypothetical protein;	111.852			127.71
ECH_0937	argininosuccinate lyase;	521.299	284.55	357.142	279.33
ECH_0938	hypothetical protein;	78.218			68.175
ECH_0939	putative polypeptide deformylase;	752.32	1184.8	276.486	1474.2
ECH_0940	dihydroorotate dehydrogenase;	1002.35	751.64	1259.55	682.34
ECH_0941	putative transcriptional activator, Baf family;	489.623	465.74	797.183	397.63
ECH_0943	hypothetical protein;	164.223	67.059	171.764	56.536
ECH_0944	Na+/H+ ion antiporter family protein;	62.0021	66.007		70.757
ECH_0945	conserved domain protein;	122.585	134.71	581.481	150.53
ECH_0946	ribonuclease HII;	106.525	128.14	410.779	126.98
ECH_0947	conserved hypothetical protein;	128.019	220.29	961.932	224.47
ECH_0950	8-amino-7-oxononanoate synthase;	119.506	184.9	175.809	209.42
ECH_0951	DNA-directed RNA polymerase, beta' subunit;	1387.07	1895.4	813.115	1937.2
ECH_0952	DNA-directed RNA polymerase, beta subunit;	1454.93	1877.5	877.628	1917.5
ECH_0953	ribosomal protein L7/L12;	2914.59	2155.8	1480.64	2384
ECH_0954	ribosomal protein L10;	929.992	678.14	602.774	755.35
ECH_0955	ribosomal protein L1;	1088.15	1275	917.283	1222.7
ECH_0956	ribosomal protein L11;	760.908	1276.9	874.293	1120.9
ECH_0957	transcription antitermination protein NusG;	1077.56	1197.6	1138.2	1206.5
ECH_0958	putative preprotein translocase, SecE subunit;	477.604	323.35	392.107	482.42
ECH_0961	translation elongation factor G;	3746.54	4763.4	2069.2	4769.9
ECH_0962	ribosomal protein S7;	3014.19	3529	1647.58	3218.1
ECH_0963	ribosomal protein S12;	8960.85	5654.6	3860.99	5383.9
ECH_0964	serine/threonine phosphoprotein phosphatase;	1281.07	1599.6	557.77	1377.1
ECH_0965	hypothetical protein;	1079.14	859.65	412.289	793.23
ECH_0966	metallo-beta-lactamase family, beta-CASP subfamily;	819.951	772.94	725.507	799.65
ECH_0967	conserved hypothetical protein;	161.553	59.257		35.19
ECH_0970	type I secretion membrane fusion protein, HlyD family;	473.752	482.89	247.155	388.08
ECH_0971	hypothetical protein;	383.624	157.46		234.6
ECH_0972	ABC transporter, permease protein;	523.992	284.65	194.176	309.585

ECH_0973	cytochrome b561 family protein;	1240.31	1565.2	1030.9	1487.5
ECH_0974	trans-2-enoyl-ACP reductase II;	616.947	675.3	568.499	681.21
ECH_0976	major antigenic protein;	1469.72	800.26	649.071	1006.5
ECH_0977	putative phosphate ABC transporter, periplasmic phosphate-binding protein;	509.165	493.05	399.603	458.84
ECH_0978	ribosomal protein S21;	3111.96	4907.9	6161.69	3437.9
ECH_0979	succinyl-CoA synthetase, beta subunit;	2524.39	3088.1	3546.15	1889.6
ECH_0980	succinyl-CoA synthetase, alpha subunit;	1653.22	2379.7	1892.52	1498.1
ECH_0981	alanyl-tRNA synthetase;	285.412	378.95	551.29	389.85
ECH_0982	phosphate ABC transporter, permease protein;	134.9	157.53	166.776	139.45
ECH_0983	glutamine synthetase domain protein;	86.966	88.369	275.654	74.087
ECH_0985	metallo-beta-lactamase family protein;	212.803	187.45	220.56	360.31
ECH_0986	hypothetical protein;	101.683	139.18	347.451	100.8
ECH_0987	hypothetical protein;	107.412			101.41
ECH_0988	conserved hypothetical protein;	138.659	54.755		72.379
ECH_0989	conserved hypothetical protein;	109.734	45.622	196.479	48.91
ECH_0991	hypothetical protein;	11.4412	19.575		14.494
ECH_0992	dihydrolipoamide dehydrogenase;	178.867	187.29	234.512	230.24
ECH_0995	hypothetical protein;	199.735	294.56		395.3
ECH_0996	ATP-dependent protease HslV;	849.591	1152.5	976.141	1264.9
ECH_0997	heat shock protein HslVU, ATPase subunit HslU;	818.885	994.42	587.759	994.79
ECH_0998	ubiquinone/menaquinone biosynthesis methlytransferase UbiE;	722.529	322.14	345.428	391.01
ECH_1000	methionyl-tRNA synthetase;	177.996	243.41	344.544	309.7
ECH_1001	aspartate kinase;	388.61	414.36	371.47	355.57
ECH_1002	cytochrome c oxidase, subunit II;	2773.63	2347.3	2256.9	1772.9
ECH_1003	cytochrome c oxidase, subunit I;	1740.27	2144.2	1371.24	1678.4
ECH_1004	protoheme IX farnesyltransferase;	717.967	919.52	1092.87	899.88
ECH_1005	putative competence protein ComL;	1496.89	1205.1	799.055	1362.1
ECH_1006	phosphoribosylamine--glycine ligase;	2030.05	2321.2	1292.93	2191.4
ECH_1008	preprotein translocase, YajC subunit;	5126.54	3620.7	3491.62	5174.5
ECH_1009	DNA polymerase III, beta subunit;	989.352	776.57	862.636	917.66
ECH_1010	DNA polymerase III, beta subunit, truncation;	81.9579	92.957		107.33
ECH_1011	3'-5' exonuclease family protein;	2253.97	2373.3	1104.59	2708.7
ECH_1012	DNA-binding response regulator;	5174.13	5428.9	1605.28	5771.7

ECH_1013	DNA-binding protein;	1323.38	1050.7	1004.29	1565.2
ECH_1014	inorganic pyrophosphatase;	1661.67	448.74	536.031	689.34
ECH_1017	N-acetyl-gamma-glutamyl-phosphate reductase;	648.341	624.82	602.851	802.84
ECH_1018	ribosomal protein S9;	4009.18	4888	2734.5	5283.6
ECH_1019	ribosomal protein L13;	8579.94	10159	7568.67	10423
ECH_1020	putative outer membrane protein TolC;	684.084	639.74	295.005	759.87
ECH_1021	conserved domain protein;	339.715	400.67	186.251	445.69
ECH_1023	conserved hypothetical protein;	691.332	659.76	916.398	715.65
ECH_1024	ribose-phosphate pyrophosphokinase;	88.455	100.84	155.491	94.634
ECH_1025	glutamyl-tRNA(Gln) amidotransferase subunit C;	556.081	588.21	385.105	614.77
ECH_1030	hypothetical protein;	52.4557	112.92		169.51
ECH_1031	aconitate hydratase 1;	784.39	907.04	987.206	929.96
ECH_1033	apaG protein;	2178.66	2696.6	4121.48	2856.3
ECH_1034	phosphoribosylaminoimidazole carboxylase, ATPase subunit;	50.5608	54.681	375.318	65.206
ECH_1036	conserved domain protein;	75.1455	130.55	666.217	113.53
ECH_1037	hypothetical protein;	1045.93	1026.5	766.71	984.64
ECH_1038	hypothetical protein;	34.0411	40.315	511.696	44.655
ECH_1041	type IV secretion system protein VirB4;	735.343	787.68	590.884	611.75
ECH_1042	conserved hypothetical protein;	2897.98	3864.9	1725.27	3245.4
ECH_1043	conserved hypothetical protein;	3065.45	4351.3	2072.02	4159.2
ECH_1044	conserved hypothetical protein;	5780.57	8465.2	4059.46	8805.4
ECH_1047	conserved hypothetical protein;	137.355	274.37	991.336	250.2
ECH_1050	hflK protein;	2012.36	1817.7	1530.09	1655.9
ECH_1051	hflC protein;	870.445	1108.1	319.77	794.36
ECH_1052	serine protease, DO/DeqQ family;	1074.46	1422	854.412	1264
ECH_1053	conserved hypothetical protein;	541.209	248.01	244.562	172.52
ECH_1054	ribonuclease III;	155.661	245.25	304.013	191.19
ECH_1055	cytochrome c oxidase assembly protein CtaG;	161.769	170.46	196.054	125.86
ECH_1057	peptidase, M16 family;	693.873	754.25	945.959	604.13
ECH_1058	peptidase, M16 family;	343.069	325.71	472.35	326.49
ECH_1059	conserved hypothetical protein;	35577.8	38370	65132.9	30658
ECH_1060	signal peptidase II;	2810.13	2036.7	4885.34	1907.4
ECH_1061	riboflavin biosynthesis protein RibF;	655.506	370.57	298.396	325.93
ECH_1062	glutaredoxin 3;	610.72	520.52	289.299	701.93

ECH_1063	modification methylase, HemK family;	201.317	106.77	330.012	157.9
ECH_1064	methionine aminopeptidase, type I;	388.453	443.67	928.868	480.46
ECH_1065	2-oxoglutarate dehydrogenase, E2 component, dihydrolipoamide succinyltransferase;	1449.3	1799.2	937.185	1965.8
ECH_1066	hexapeptide transferase family protein;	263.128	318.34	441.407	398.28
ECH_1067	D-alanyl-D-alanine carboxypeptidase family protein;	582.949	475.27	389.541	442.75
ECH_1068	C-type cytochrome family protein;	928.766	614.78	996.164	507.58
ECH_1069	TPR repeat-containing protein	107.846	199.95		171.93
ECH_1070	putative membrane-associated zinc metalloprotease;	2747.59	2660.2	1754.71	2437.4
ECH_1071	outer membrane protein, OMP85 family;	1104.85	991.01	671.496	835.19
ECH_1072	outer membrane protein, OmpH family;	2054.5	1399.4	1414.16	1821.9
ECH_1073	beta-hydroxyacyl-(acyl-carrier-protein) dehydratase FabZ;	1302.82	942.91	688.911	1218.7
ECH_1074	phosphoribosylaminoimidazolecarboxamide formyltransferase/IMP cyclohydrolase;	337.77	387.69	251.958	385.53
ECH_1077	hypothetical protein;	158.42			52.335
ECH_1078	CDP-diacylglycerol--glycerol-3-phosphate 3-phosphatidyltransferase;	566.191	249.54	502.387	296.13
ECH_1079	putative cytochrome c oxidase, subunit I;	98.6305	105.72	254.009	94.891
ECH_1081	SURF1 family protein;	310.72	78.565	255.52	80.876
ECH_1083	pentapeptide repeat protein;	163.898	94.619	170.257	77.14
ECH_1084	AraM protein;	684.787	364.76	554.552	307.48
ECH_1086	ATP synthase F0, A subunit;	207.326	244.64	167.931	232.46
ECH_1087	ATP synthase F0, C chain;	2617.66	11452	11016.1	13306
ECH_1088	ATP synthase F0, B' subunit;	7563.08	6857.5	10489.4	6985.3
ECH_1089	ATP synthase F0, B chain;	3119.55	2800.8	3288.13	2852.2
ECH_1090	cell division protein FtsA;	1157.38	1236.4	1124.95	1049.6
ECH_1093	putative potassium uptake protein TrkH;	973.254	320.38	416.262	217.48
ECH_1097	tRNA(Ile)-lysidine synthetase;	264.143	260.94	277.63	308.47
ECH_1098	ATP-dependent metalloprotease FtsH;	730.589	837.27	878.872	688.4
ECH_1101	prolipoprotein diacylglyceryl transferase;	1143.89	190.28	598.626	148.02
ECH_1103	conserved hypothetical protein;	1170.04	758.04	1261.22	502.41
ECH_1104	hypothetical protein;	767.823	1125.6	974.073	807.1
ECH_1105	conserved domain protein;	254.208	331.38		372.75
ECH_1106	protein-export membrane protein SecD;	1128.36	958.44	788.477	864.01

ECH_1107	adenylyltransferase thiF;	357.45	580.66	388.351	651.97
ECH_1108	orotate phosphoribosyltransferase;	1983.7	1371.7	938.117	1564.6
ECH_1109	recA protein;	3967.21	4350.8	1463.83	4477
ECH_1110	dethiobiotin synthetase;	151.865	116.49	196.054	152.99
ECH_1111	signal recognition particle-docking protein FtsY;	261.565	262.55	318.981	256.73
ECH_1113	hypothetical protein;	233.457	220.92		179.75
ECH_1114	dehydrogenase, isocitrate/isopropylmalate family;	1767.35	2008.8	919.788	1791.4
ECH_1115	single-stranded-DNA-specific exonuclease RecJ;	316.683	354.9	332.627	316.85
ECH_1116	polyA polymerase/tRNA nucleotidyltransferase family protein;	138.487	86.94	257.503	94.321
ECH_1117	nucleoside diphosphate kinase;	2622.08	2599.8	1689.08	2660.8
ECH_1118	putative transcriptional regulator;	388.44	403.24	1279.85	244.27
ECH_1119	major outer membrane protein OMP-1M;	1260.84	1404.7	1269.43	1500.3
ECH_1121	major outer membrane protein Omp-1N;	1578.02	4412.6	3132.73	4612.4
ECH_1122	conserved hypothetical protein;	4427.32	701.62	1244.74	1085.7
ECH_1123	major outer membrane protein OMP-1Q;	7709	4795.1	3090.15	4430.1
ECH_1124	major outer membrane protein OMP-1P;	5166.83	3905.1	2390.35	3696.3
ECH_1125	major outer membrane protein OMP-1T;	2601.68	1913.5	1335.03	1631
ECH_1126	major outer membrane protein OMP-1U;	3891.28	3286.2	1726.73	3171.8
ECH_1127	major outer membrane protein OMP-1V;	6579.46	5283.4	3412.03	6190.1
ECH_1128	conserved hypothetical protein;	1851.72	1004.8	1806.28	1282.5
ECH_1129	major outer membrane protein OMP-1W;	2727.37	2482	1093.48	2668.4
ECH_1130	major outer membrane protein OMP-1X;	4659.57	4788.4	1656.51	5178.3
ECH_1131	major outer membrane protein OMP-1Y;	4593.53	5734.9	1485.48	6128.6
ECH_1132	major outer membrane protein OMP-1S;	4053.41	4361.9	1506.66	5079.3
ECH_1133	major outer membrane protein OMP-1H;	4222.07	3784.9	1456.96	4540.2
ECH_1134	major outer membrane protein OMP-1Z;	4520.01	5195.3	1698.05	5628.3
ECH_1135	major outer membrane protein OMP-1A;	3926.4	3780.9	1804.49	4680.9
ECH_1136	major outer membrane protein OMP-1B;	698.247	969.26	8269.46	859.52
ECH_1137	major outer membrane protein OMP-1C;	2718.49	2735.2	1557.96	2832.3
ECH_1139	major outer membrane protein OMP-1D;	545.71	1131.6	285.542	1044.8
ECH_1140	major outer membrane protein OMP-1E;	2795.38	2487.6	602.918	3010.8
ECH_1142	major outer membrane protein OMP-1F;	2743.82	2257.5	775.144	2612.1
ECH_1143	major outer membrane protein P28;	3957.1	22761	8358.7	30614
ECH_1144	major outer membrane protein P28-1;	6874.84	6480.4	3853.32	7730.5

ECH_1146	major outer membrane protein P28-2;	190.857	141.9	1100.64	157.76
ECH_1147	conserved hypothetical protein;	1982.05	5528.3	2632.06	4756.9
ECH_1148	conserved hypothetical protein;	5187.27	5543.5	3800.42	6364.2
ECH_1149	preprotein translocase, SecA subunit;	609.747	685.24	752.183	498.4
ECH_1150	hypothetical protein;	213.766	161.68		220.69
ECH_1151	hypothetical protein;	472.828	278.36	1552.74	252.34
ECH_1152	hypothetical protein;	3163.94	3849.5	7733.58	4380.7
ECH_1153	cell division protein FtsZ;	4634.79	5167.1	2417.22	5269.5
ECH_1154	conserved hypothetical protein;	362.621	127.36	222.002	180.2
ECH_1155	conserved hypothetical protein TIGR00043;	137.765	49.886	236.529	67.336
ECH_1156	chromosome partitioning ATPase, ParA family;	105.838	110.72	201.394	104.18
ECH_0068	hypothetical protein;	190.584	113.9640612	58.514714	69.12354
ECH_0610	hypothetical protein;	0.56099	0.733972196		111.1987
ECH_0819	hypothetical protein;	2.96993	2.590490103		104.3906
ECH_1138	hypothetical protein;	2.1037			142.0873
ECH_0017	hypothetical protein;	7.3886	2.148211305		
ECH_0047	hypothetical protein;	11.921	7.339721959	6.4435846	
ECH_0051	hypothetical protein;	66.671	30.06463033	35.687546	
ECH_0053	hypothetical protein;	15.465	5.356013322	16.71849	
ECH_0054	hypothetical protein;	19.7029	18.25979609	30.174835	
ECH_0055	hypothetical protein;	20.8687	11.89034957	12.371682	
ECH_0059	hypothetical protein;	20.8687	11.89034957	12.371682	
ECH_0070	hypothetical protein;	12.3143	5.370528263	7.5437088	
ECH_0075	hypothetical protein;	2.40423	0	7.9305657	
ECH_0083	hypothetical protein;	0.96169	2.5164761		
ECH_0094	hypothetical protein;	5.01307	5.153421801	25.248332	
ECH_0102	hypothetical protein;	1.08578	1.775739184	6.8731569	
ECH_0104	hypothetical protein;	1.60282	0		
ECH_0110	hypothetical protein;	7.7675	3.952157978	6.5806822	
ECH_0111	hypothetical protein;	6.36795	5.951125913		
ECH_0112	hypothetical protein;	1.34637	1.76153327		
ECH_0182	hypothetical protein;	0.82096	0		
ECH_0216	hypothetical protein;	1.81941	2.380450365	17.673832	
ECH_0241	hypothetical protein;	1.34637	0.440383318	3.188578	
ECH_0242	hypothetical protein;	2.49327	4.485385642	6.1858412	
ECH_0244	hypothetical protein;	0.88577	1.158903467		
ECH_0245	hypothetical protein;	2.80493	4.281504476		
ECH_0262	hypothetical protein;	2.46287	0		

ECH_0265	hypothetical protein;	3.13109	2.0482945		
ECH_0268	hypothetical protein;	0.68692	0.898741464		
ECH_0273	hypothetical protein;	2.14846	5.153421801		
ECH_0283	hypothetical protein;	2.14846	1.405478673		
ECH_0286	hypothetical protein;	5.74669	1.074105653	17.182892	
ECH_0358	hypothetical protein;	0.84148	0.550479147	8.836916	
ECH_0361	hypothetical protein;	1.74099	0.379640791		
ECH_0375	hypothetical protein;	0.80141			
ECH_0456	hypothetical protein;	0.88577			
***Ehrlichia chaffeensis* genes mapped to < 10 reads.**					
ECH_0457	hypothetical protein;	3.73991	0.489314797		
ECH_0506	hypothetical protein;	2.08201	2.497018811		
ECH_0524	hypothetical protein;	1.60282	1.572797563		
ECH_0582	hypothetical protein;	4.2074	1.032148401		
ECH_0598	hypothetical protein;	0	0.611643497		
ECH_0606	hypothetical protein;	0.56099	0.366986098		
ECH_0608	hypothetical protein;	2.01955	0.880766635		
ECH_0658	hypothetical protein;	1.46344	0.957355038	6.1858412	
ECH_0661	conserved hypothetical protein;	6.1823	4.04433659		
ECH_0672	hypothetical protein;	1.71981	1.446514547		
ECH_0711	hypothetical protein;	2.34831	1.02414725		
ECH_0717	hypothetical protein;	0	1.785337774		
ECH_0719	hypothetical protein;	0.82096	0.537052826		
ECH_0736	hypothetical protein;	1.46344	0.478677519		
ECH_0749	hypothetical protein;	1.60282	2.097063417		
ECH_0867	hypothetical protein;	0	0.772602312	7.7323015	
ECH_0887	hypothetical protein;	0	0.579451734	8.3592449	
ECH_0968	hypothetical protein;	3.9599	1.295245052		
ECH_0969	hypothetical protein;	3.36592	3.082683223		
ECH_1022	hypothetical protein;	5.98385	0.489314797		
ECH_1027	hypothetical protein;	0.84148	0.550479147		
ECH_1029	hypothetical protein;	3.36592	3.963449858		
ECH_1056	hypothetical protein;	2.99193	3.914518378		
ECH_1082	hypothetical protein;	0	2.90819172		
ECH_1091	hypothetical protein;	1.86995	0.611643497		
ECH_1092	hypothetical protein;	2.88507	3.145595125		

Table S2. The top 100 highly expressed genes in transcriptome of wildtype *E. chaffeensis*		
Gene ID	Product	Wildtype RPKM
ECH_0166	conserved hypothetical protein;	42488.4
ECH_1059	conserved hypothetical protein;	35577.8
ECH_0170	variable length PCR target protein;	17918.5
ECH_0689	iron-sulfur cluster assembly accessory protein;	17663.3
ECH_0308	ribosomal protein S6;	14180.2
ECH_0570	hypothetical protein;	13247.1
ECH_0227	malonyl CoA-acyl carrier protein transacylase;	11175.8
ECH_0253	hypothetical protein;	10952.4
ECH_0252	hypothetical protein;	9827.77
ECH_0963	ribosomal protein S12;	8960.85
ECH_0079	hypothetical protein;	8953.79
ECH_1019	ribosomal protein L13;	8579.94
ECH_0697	hypothetical protein;	8509.63
ECH_0298	cold shock protein, CSD family;	8294.62
ECH_0876	ribosomal protein L33;	8272.92
ECH_1123	major outer membrane protein OMP-1Q;	7709
ECH_0484	50S ribosomal protein L28;	7585.98
ECH_1088	ATP synthase F0, B' subunit;	7563.08
ECH_0432	DNA-directed RNA polymerase, alpha subunit;	7468.67
ECH_0430	ribosomal protein S13;	7384.34
ECH_0009	putative membrane protein;	7047.64
ECH_1144	major outer membrane protein P28-1;	6874.84
ECH_0364	chaperonin, 10 kDa;	6801.56
ECH_0707	hypothetical protein;	6790.2
ECH_1127	major outer membrane protein OMP-1V;	6579.46
ECH_0309	putative ribosomal protein S18;	6545.87
ECH_0071	ribosomal protein S20;	6265.18
ECH_0365	chaperonin, 60 kd;	6017.96
ECH_1044	conserved hypothetical protein;	5780.57
ECH_0199	conserved hypothetical protein;	5628.22
ECH_0690	signal peptidase I;	5614.13
ECH_0122	hypothetical protein;	5514.52
ECH_0172	preprotein translocase, SecG subunit;	5505.56

ECH_0676	arginine biosynthesis bifunctional protein ArgJ;	5470.9
ECH_0310	ribosomal protein L9;	5385.81
ECH_0912	ribosomal protein S4;	5230.68
ECH_1148	conserved hypothetical protein;	5187.27
ECH_1012	DNA-binding response regulator;	5174.134
ECH_1124	major outer membrane protein OMP-1P;	5166.83
ECH_1008	preprotein translocase, YajC subunit;	5126.54
ECH_0734	antioxidant, AhpC/Tsa family;	4875.62
ECH_0681	hypothetical protein;	4787.59
ECH_0431	ribosomal protein S11;	4707.86
ECH_1130	major outer membrane protein OMP-1X;	4659.57
ECH_0862	hypothetical protein;	4653.15
ECH_1153	cell division protein FtsZ;	4634.79
ECH_1131	major outer membrane protein OMP-1Y;	4593.53
ECH_0198	ribosomal protein L35;	4522.63
ECH_1134	major outer membrane protein OMP-1Z;	4520.01
ECH_1122	conserved hypothetical protein;	4427.32
ECH_0573	ATP synthase F1, beta subunit;	4397.11
ECH_0233	protein-export protein SecB;	4262.32
ECH_1133	major outer membrane protein OMP-1H;	4222.07
ECH_0520	ubiquinol-cytochrome c reductase, iron-sulfur subunit;	4210.67
ECH_0197	ribosomal protein L20;	4187.32
ECH_0147	conserved hypothetical protein;	4121.06
ECH_0702	folylpolyglutamate synthase;	4118.64
ECH_1132	major outer membrane protein OMP-1S;	4053.41
ECH_1018	ribosomal protein S9;	4009.18
ECH_0727	ribosomal protein S15;	3986.2
ECH_1109	recA protein;	3967.21
ECH_1143	major outer membrane protein P28;	3957.1
ECH_1135	major outer membrane protein OMP-1A;	3926.4
ECH_0462	OmpA family protein;	3901.23
ECH_0193	major facilitator family transporter;	3894.26
ECH_1126	major outer membrane protein OMP-1U;	3891.28
ECH_0811	enoyl-(acyl-carrier-protein) reductase;	3875.96
ECH_0726	polyribonucleotide nucleotidyltransferase;	3812.81
ECH_0004	ribosomal protein L19;	3780.85
ECH_0961	translation elongation factor G;	3746.54
ECH_0443	dihydrodipicolinate reductase;	3714.48
ECH_0010	hypothetical protein;	3668.83

ECH_0192	ribosomal protein S16;	3521.36
ECH_0627	conserved hypothetical protein;	3437.62
ECH_0825	hypothetical protein;	3431.48
ECH_0444	phosphate ABC transporter, ATP-binding protein;	3422.81
ECH_0705	peptide chain release factor 2, programmed frameshift;	3364.17
ECH_0638	ribose 5-phosphate isomerase B;	3274
ECH_0311	serine hydroxymethyltransferase;	3271.5
ECH_0858	DNA gyrase, A subunit;	3263.43
ECH_0779	phosphatidylserine decarboxylase;	3257.68
ECH_0886	acetylornithine/succinyldiaminopimelate aminotransferase;	3243.91
ECH_1152	hypothetical protein;	3163.94
ECH_1089	ATP synthase F0, B chain;	3119.55
ECH_0804	DNA-binding protein HU;	3113.23
ECH_0978	ribosomal protein S21;	3111.96
ECH_1043	conserved hypothetical protein;	3065.45
ECH_0744	conserved hypothetical protein;	3042.46
ECH_0962	ribosomal protein S7;	3014.19
ECH_0433	ribosomal protein L17;	3003.07
ECH_0896	rhodanese domain protein;	2973.13
ECH_0471	chaperone protein DnaK;	2933.79
ECH_0953	ribosomal protein L7/L12;	2914.59
ECH_0929	putative lipoprotein;	2898.72
ECH_1042	conserved hypothetical protein;	2897.98
ECH_0008	P-loop hydrolase family protein;	2879.4
ECH_0142	ribosomal 5S rRNA E-loop binding protein Ctc/L25/TL5;	2875.81
ECH_0888	putative membrane protein;	2867.5
ECH_0652	ATP synthase F1, gamma subunit;	2852.18
ECH_0377	conserved domain protein;	2827.77

Table S3: Primers used for qRT-PCR				
primer name	gene target	gene name	sequence	amplicon size (bp)
RG338	ECH_1146	OMP-p28-2	F-CCCACACAAGAAAAAGGAACA	147
RG339			R-GCACATTGCTTTAGTCAGATCC	
RRG1061	ECH_1143	OMP-p28	F-AGGCGAAGGCATACCTTTTT	156
RRG1062			R-TGCCCACCAATAAACACAGA	
RG320	ECH_1136	Omp1B	F- AACGACAGCAGAGAAGGC	182
RG321			R- AACAGGACAGATCCAGCC	
RG322	ECH_1121	Omp1N	F-TCATGTTTAGGATTTGGAGTA	220
RG323			R- GTGGCTTGCTGTTTGGT	
RRG1009	ECH_0367	ClpB	F-TCTTGGAATGCTTGTTGCTG	196
RRG1010			R-ACCCCTTGCTAAAGCAGGTT	
RRG1027	ECH_0655	rpoH	F-AAAATGGGATCGTTGCTGAC	170
RRG1028			R-AAAGCCAAGAGTGGGATTGA	
RRG2054	ECH_0466	ECH_0466	F-TGCTGCAAATTTGTTTGGAA	165
RRG2055			R-TCTCCAAAAGAACCATGAAGA	
RRG2058	ECH_0469	mrpC	F-GGTTATAGGTTTGTATGTTACT	213
			ACTGC	
RRG2059			R-ATTGCAACCCCAACAACAAT	
RRG985	ECH_0085	ECH_0085	F-GGAAAATTGCTGCTTCTGGA	162
RRG986			R-AGCCTGAAGTTGGTTCATCC	
RG332	ECH_0972	ECH_0972	F- GCCCCATCAATAAAGACGAC	135
RG333			R- TTGCTCTGATTGGGACAACA	
RG334	ECH_1093	ECH_1093	F-ATGACATCCCAGCAATCCAA	168
RG335			R-TGCCTTGCTTGAAAGTTGTG	
RG330	ECH_0299	ECH_0299	F- CAGCTGCATGGTCTGATGTT	167
RG331			R- CGTCACATGGCGAATTATTG	
RG328	ECH_0033	ECH_0033	F- GCAATGTCACGTGCTGAACT	122
RG329			R- GGTCCAAGAAATGCTGGAAG	
**Primers used for Semi quantitative RT-PCR**				
RG627	ECH_0490	lipA	F- AGGTTGCTGATGCTGCTCCTGATG	310
RG628			R- ACTAAAGGGCCCGACGCAACC	
RG629	ECH_0492	ECH_0492	F- ACGGATGGTGGCACTGCTGC	271
RG630			R- TCGCATTACCTATAGGTATCACAT CCG	

Original image for Fig S1 lane 4 of panel B

ECH_0490

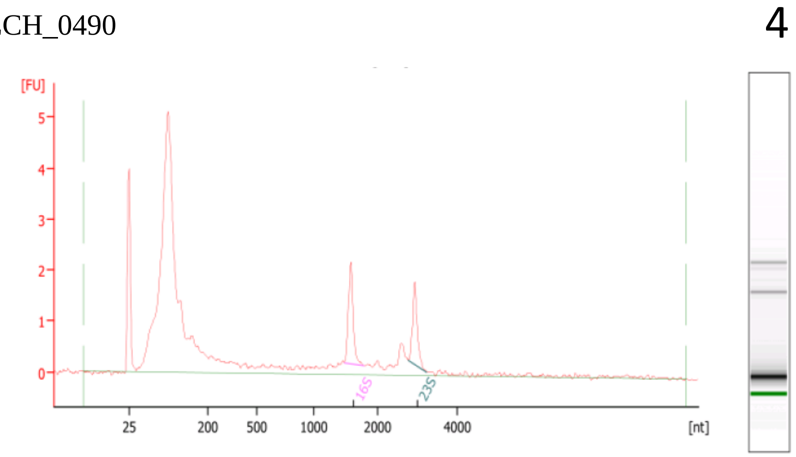

RNA ladder

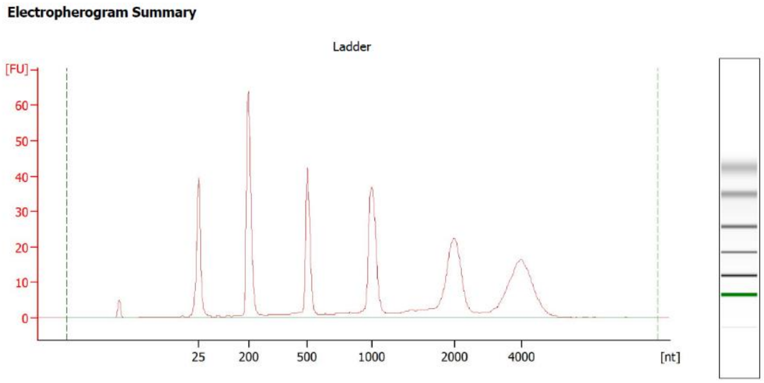

Supplement: Supplementary file 1 — Supplementary Figures and Tables [file 41598_2018_24471_MOESM1_ESM.pdf]
